# Integrating genomic epidemiology and deep mutational scanning data for prevalence forecasting of SARS-CoV-2 Omicron lineages

**DOI:** 10.1371/journal.pone.0335520

**Published:** 2025-11-03

**Authors:** Zhong-yi Lei, Xiao-min Zhang, Jia-lu Han, Ji-guo Xue, Jia-yi Xu, Zi-lin Ren, Yi-gang Tong, Xiao-chen Bo, Ming Ni

**Affiliations:** 1 College of Life Science and Technology, Beijing University of Chemical Technology, Beijing, China; 2 Advanced & Interdisciplinary Biotechnology, Academy of Military Medical Sciences, Beijing, China; 3 Changchun Veterinary Research Institute, Chinese Academy of Agricultural Sciences, State Key Laboratory of Pathogen and Biosecurity, Key Laboratory of Jilin Province for Zoonosis Prevention and Control, Changchun, China; 4 School of Information Science and Technology, Northeast Normal University, Changchun, China; Waseda University: Waseda Daigaku, JAPAN

## Abstract

Severe acute respiratory syndrome coronavirus 2 (SARS-CoV-2) continuously circulates and the Omicron variants have mutated into over 2,500 lineages, predicting ensuing prevalent lineages and inflections of dominant lineages is of public health significance and study interest. Previous study has integrated genome to forecast lineage prevalence, yet overlooked the functional aspects of mutations; efforts to evaluate the functional effects of individual mutations have not extended to the lineage level. Here, we propose CoVPF, a model integrating both genomic epidemiology and deep mutational scanning (DMS) data for the receptor binding domain (RBD) of SARS-CoV-2 spike protein, to predict the prevalence of Omicron lineages. Retrospective validation demonstrated that CoVPF achieved 20.7% higher accuracy compared to previous study. Furthermore, we found that accounting for epistasis was critical, as ignoring epistasis led to a 43% decrease in forecasting accuracy. Case studies showed that CoVPF delivered more accurate and timely forecasts for lineage expansions and inflections such as EG.5.1 and XBB.1.5. CoVPF provides a paradigm for integrating *in vitro* functional readouts of the virus and accounting for combinatorial effects of mutations in support of public health efforts in lineage prevalence forecasting.

## Introduction

On May 5, 2023, the World Health Organization (WHO) declared the end of the emergency phase of the COVID-19 pandemic, which had been designated a Public Health Emergency of International Concern for over three years. From the first case in December 2019 [1] to May 2023, over 765 million confirmed cases and 6.9 million deaths had been reported to WHO [2]. Similar to most RNA viruses, the etiologic agent, severe acute respiratory syndrome coronavirus 2 (SARS-CoV-2), continues to evolve rapidly, with novel substitutions emerging on timescales of months or years which are often observable and measurable [3]. Consequently, SARS-CoV-2 continues to circulate with a significant incidence rate within the human population due to constant antigenic evolution that evades herd immunity. From January 2024 to December 2024, an average of 277,083 incident cases and 5,434 deaths per month were reported to WHO.

During the four years of transmission and adaptation in human and animal populations [3], the prototypic SARS-CoV-2 strain, Wuhan-hu-1 (Global Initiative on Sharing All Influenza Data [GISAID] accession EPI_ISL_402125, NCBI GenBank accession NC_045512) has given rise to highly diverse descendants that are assigned to more than 2000 phylogenetic Pango lineages [4–6]. Based on biologically significant traits such as increased transmissibility, immune escape, and reduced susceptibility to treatments, multiple variants of concern (VOCs) [7] have been defined. These include Alpha (B.1.1.7 by Pangolin nomenclature), Beta (B.1.351), Gamma (P.1), Delta (B.1.617.2), and Omicron (B.1.1.529) and its descendants.

Omicron variants were first detected in November 2021 [8] and rapidly replaced regionally circulating Delta variants [9]. Compared with previous VOCs, Omicron descendants became highly prevalent and diverged into numerous lineages during the following two years. By December 2023, 1824 Omicron lineages were recorded in the GISAID database [10], accounting for 86.8% of all samples. The highly dynamic genomes [11] of Omicron variants circulating within the human population constituted a complex “variant soup” [12]*.* Because new Omicron lineages continue to emerge and usually co-circulate, anticipation of the next epidemic lineages and forecasts of the inflection points of circulating variants are of crucial public health and translational research significance.

Besides, Omicron variants exhibit significantly more amino acid mutations in the spike (S) protein than other VOCs. Compared with Wuhan-hu-1, the ancestral Omicron lineage B.1.1.529 featured 37 S protein mutations [13], compared to 9 of Delta and 6 of Alpha [14]. Moreover, Omicron exhibited 15 mutations harbored in the receptor binding domain (RBD), 11 of which were first observed in human clinical samples [11]. Numerous mutations contribute to viral fitness. For instance, the D614G substitution has been shown to enhance viral infectivity and virion stability [15], while the P681R mutation promotes spike protein cleavage and accelerates membrane fusion with host cells [16]. Via deep mutational scanning (DMS) approaches, the effects of single RBD mutations on angiotensin-converting enzyme 2 (ACE2) receptor and monoclonal antibody (mAb) binding affinities have been assessed comprehensively [17–23]. Epistasis is also remarkable for Omicron lineages [24–26]. For example, the combination of N501Y and Q498R increases S-ACE2 binding affinity to 600-fold that of wild-type [11]; whereas binding is increased 7-fold by N501Y alone and reduced by Q498R [15,24,27].

Conventional viral prevalence forecasting typically relies on system dynamics models, such as the susceptible–exposed–infectious–recovered (SEIR) framework. Volz et al. employed the SEIR model to estimate the selection coefficient of the D614G mutation in the United Kingdom [28], and Tian et al. applied a system dynamics approach to evaluate the impact of China’s emergency response on containing the COVID-19 epidemic [29]. Nevertheless, system dynamics models have limited capacity to incorporate genomic data and face challenges due to the need for accurately setting initial population values. To address these limitations, Obermeyer et al. proposed the PyR0 model, which can integrate genomics and epidemiologic spatio-temporal distributions to analyze the viral fitness of SARS-CoV-2 lineages and forecast lineage prevalence [30]. They found that the inclusion of genomics data enables PyR0 to infer elevated fitness of lineages faster. Furthermore, functional data provide additional insight beyond genomic mutations, as mentioned above, the rise of highly prevalent variants is largely driven by their functional impact. Theoretically, incorporating such information should further enhance forecasting performance. However, to the best of our knowledge, no prevalence forecasting method has systematically incorporated and validated the functional effects of mutations, such as those measurable by *in vitro* DMS.

Previous applications of DMS data have predominantly focused on predicting the functional impact of individual mutations, estimating their contribution to viral fitness, or identifying potential driver mutations, rather than forecasting the prevalence of viral lineages. For example, Wang et al. developed a model called UniBind to predict the effects of mutations on receptor binding affinity and immune escape [31], and Han et al. proposed a framework named MLAEP to identify immune escape mutations in prevalent SARS-CoV-2 variants such as XBB.1.5 [32]. Fabrizio et al. used SpikePro to evaluate fitness through linear multiplication of computationally predicted functional metrics of mutations, which might overlook epistasis [33]. Maher et al. developed a model that integrated 15 features of epidemiology, evolution, transmissibility, and resistance to population-level host immunity to predict the next drivers of S protein mutations [34], but did not extend the forecast to the lineage level.

Motivated by these limitations, we propose a model, named SARS-CoV-2 Omicron Prevalence Forecast (CoVPF), to address the issue of lineage-level prevalence forecasting by utilizing multimodal data that include genomic variation, the spatial-temporal distributions of variants, and DMS functional data. In addition, we validated the performance of CoVPF in forecasting the prevalence of Omicron lineages by comparing its results with real-world GISAID data and the results of other models. We suggest that CoVPF offers a unique *in-silico* platform for the integration of multimodal genomic and epidemiologic data, and provides a practical tool for public health purposes.

## Results

### Epidemiology of omicron variants and S protein mutations

As of December 19, 2023, genome sequences and metadata of 16.3 million SARS-CoV-2 samples were deposited into the GISAID database. A total of 8.8 million genomes were assigned to Omicron lineages, nearly twice that of Delta (4.6 million) and much more than other VOCs (S1A Fig). Omicron descendants exhibited high phylogenetic diversity; 86.7% (*n* = 1824) of Pangolin nomenclature lineages were from Omicron, which were 7.44-fold of Delta lineages (*n* = 245) (S1B Fig). Based on GISAID genomic surveillance, the first global take-over of COVID-19 by Omicron occurred in January 2022 (S1C Fig). From November 2021 to December 2023, 12 globally dominant Omicron lineages emerged (S1D Fig).

Among 215 countries and regions, the USA and the UK contributed the most SARS-CoV-2 genomic surveillance data (30.5% and 19.1%, respectively), followed by Germany (5.8%) and Japan (4.0%) (S1E Fig). The temporal patterns of regionally dominant Omicron lineages were somewhat similar, but specific lineages diverged in scale and emergence times (S2 Fig). For example, BA.2 become dominant (>50% frequency) in March to June 2022 in several European countries; whereas in the USA, BA.2 peaked at 46.9% on April 6, 2022 and was rapidly surpassed by BA.2.12.1 in May 2022.

Using the prototypic variant as the reference strain, 558 amino acid mutations were identified in the S protein of Omicron variants, of which 325 (58.2%) were specific for Omicron. Mutational diversity was apparently higher in the RBD and N-terminal domain (NTD) than in other S protein domains (S1F Fig); 92% of high-diversity (Shannon diversity index ≥ 0.1) positions were harbored in the RBD and NTD. We further assessed minor allele frequencies (MAFs) of RBD and NTD mutations, and found that RBD had significantly more mutations with fewer MAFs (<0.025) than those harbored in NTD (Kolmogorov-Smirnov test, *P* = 6.36 × 10^−9^) (S1G Fig), indicating a stronger purifying selection pressure on RBD than NTD.

### CoVPF design and framework

**Fig 1:**
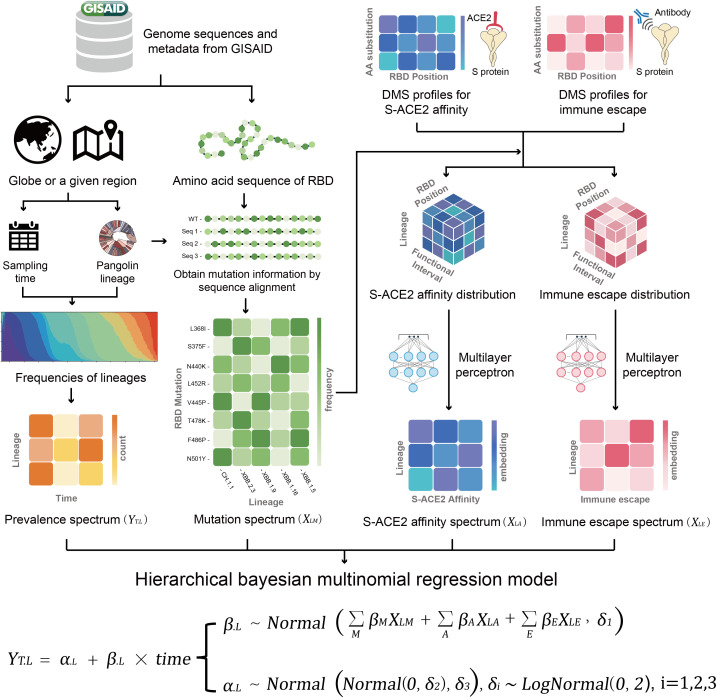
Schematic diagram of construction and framework of SARS-CoV-2 Omicron Prevalence Forecast (CoVPF). DMS, deep mutational scanning. After the construction of the four spectra, the hierarchical Bayesian multinomial regression model could be trained to obtain the regression parameters *α*_*.L*_ and *β*_*.L*_ for further fitting or forecast. See methods for details.

Since the continuing circulation of SARS-CoV-2 Omicron variants with diverse genome composition, we aimed to develop a tool to forecast the prevalence of Omicron lineages during ensuing weeks. We obtained the genomic epidemiology data of Omicron variants and *in vitro* DMS data for S-ACE2 and S-mAb affinity [17–21] for single amino acid substitutions in the RBD from public databases (methods). We utilized these multimodal data as illustrated in Fig 1. Within a global or regional location, a matrix of lineage variant counts over time was generated based on GISAID metadata, which we designated as the prevalence spectrum (denoted as *Y*_*T.L*_). In the prevalence spectrum, we adapted a non-overlapping 4-day time interval, considering the regularity of data submissions to GISAID. We only included RBD mutations, because they are the most functionally relevant, and matched the DMS data. We constructed a mutation spectrum (*X*_*LM*_) consisting of mutational frequencies in each lineage. We discretized DMS data (S-ACE2 affinity and immune escape respectively) into intervals and constructed 3-dimensional tensors (lineage, site, and functional interval) with the corresponding within-lineage mutational frequencies derived from the mutation spectrum. Then, given the absence of prior studies considering epistasis, we explored a preliminary approach using a multilayer perceptron (MLP) to transform single-site functional data along the site dimension into combinatorial embeddings, generating two 2-dimensional spectra respectively for S-ACE2 affinity (*X*_*LA*_) and immune escape (*X*_*LE*_).

We used a hierarchical Bayesian multinomial logistic regression model to integrate the four spectra (*Y*_*T.L*_*, X*_*LM*_*, X*_*LA*_ and *X*_*LE*_). With an a priori assumption of normality, the distributions of the regression parameters were continuously learned by using variational inference, in which the prevalence spectrum was the observed variable (likelihood) and the other three spectra entered as covariates affecting the parameters (Methods, S3 Fig, S2 Table). In this statistical framework, the fitness of viral lineages and mutations were represented by the *β*_*.L*_ and *β*_*M*_ parameters, respectively, similar to the study by Obermeyer et al [30]. Forecasts are then generated by applying the learned parameters to the multinomial logistic linear regression to compute predicted prevalences across lineages for future time intervals.

### Evaluation of CoVPF performance

Due to the current lack of predictive methods, for comparison, we built three control models for CoVPF. The first (denoted as CM_noDMS_) disregarded DMS functional data (*X*_*LA*_ and *X*_*LE*_). This approach was similar to that used in the PyR0 model; however, PyR0 includes mutations across the whole genome instead of only the RBD [30]. For the second control model (CM_ranDMS_), we used randomly generated values to replace the real DMS dataset. The third control model (CM_noEpis_) used a linear layer to replace the MLP, and thus the epistasis could not be considered.

We first evaluated CoVPF performance on prevalence fitting of the continuously emerging Omicron lineages. We constructed the prevalence spectrum *Y*_*T.L*_ and mutation spectrum (*X*_*LM*_), based on global data from November 1, 2021 to December 19, 2023, as well as the two DMS spectra. The model fitting of prevalence is shown in S4A Fig. CoVPF outperformed all three controls and PyR0, with lower mean absolute error (MAE) and mean square error (MSE) between fitted and real-world prevalence proportions (S4B-C Figs). Interestingly, the performance of CoVPF and CM_ranDMS_ were comparable, and greatly surpassed those of PyR0, CM_noDMS_, and CM_noEpis_, implying that taking epistasis into account might be more critical than the initial DMS values.

**Fig 2:**
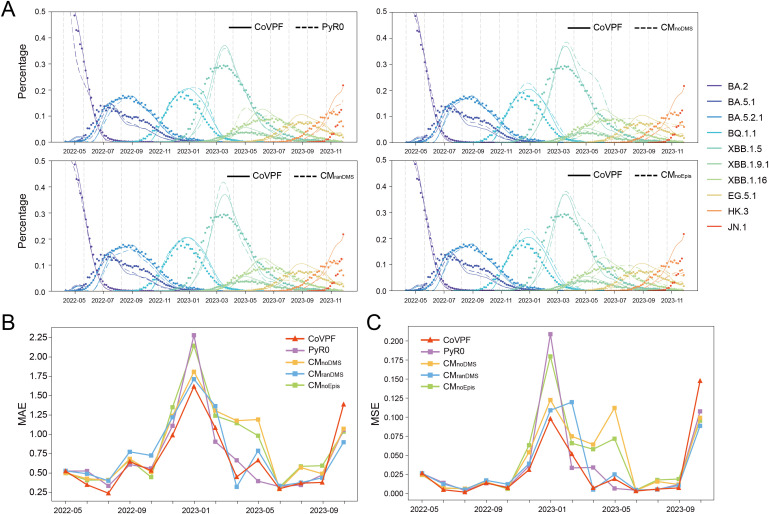
Evaluation of CoVPF performance and comparison with PyR0 and three control models. **(A)** Global forecasts of prevalence in 40-day increments from April 2022 to December 2023. The time points when the forecasts were made are denoted by vertical dashed lines. CM_noDMS_, the control model that ignored deep mutational scanning (DMS) data. CM_ranDMS_, the control model using random values for DMS. CM_noEpis_, the control model did not consider epistasis. **(B-C)** The performance of forecasting with time. Mean absolute errors (MAEs, B) and mean squared errors (MSEs, C) were used to quantified the performance of forecasts corresponding to that denoted in A.

Next, we evaluated how CoVPF performed in forecasting the global prevalence of Omicron lineages. The initial training datasets covered 5.5 months (November 1, 2021 to April 14, 2022), during the circulations of BA.1, BA.2, and BA.3. We conducted forecasts for 40-day increments (Fig 2A, indicated as vertical dashed lines). Each forecast comprised the predicted lineage prevalence rates at ten time points (the next 40 days, with 4-day bins). Training datasets were continuously expanded with previous 40 days data to update the input. Parallel forecasting was conducted by using the control models. MAEs and MSEs for CoVPF and control forecasts are shown in Fig 2B-C. The accumulated MAEs and MSEs of CoVPF for all forecasts were 9.46 and 0.424, respectively, which were smaller than those of PyR0 (10.05 and 0.512) and CM_ranDMS_ (10.31 and 0.479). CM_noDMS_ and CM_noEpis_ both exhibited large biases in prevalence prediction compared to real data, with ~11.6 MAE and ~0.61 MSE.

The forecasting performance of all models fluctuated over time. The superiority of CoVPF compared to CM_noDMS_ and CM_noEpis_ seemed increasingly significant as the training set expanded, while its superiority to CM_ranDMS_ was limited to the early stage (before December 2022) (Fig 2B-C). Two additional independently randomized controls performed similarly to CM_ranDMS_, with advantage of CoVPF confined to early forecast periods (MAE, MSE in S18 Fig). Meanwhile, the weight matrices were mutually uncorrelated between CoVPF and all randomized controls (S19 Fig). Notably, the forecasting accuracies of CoVPF and other models exhibited transient yet substantial declines from December 2022 to March 2023, with PyR0 exhibiting a particularly pronounced decrease. In October 2023, the decline recurred.

We also assessed model performance on fitting and forecasting in the USA, the UK, Germany, France, Brazil, and Poland. Results were qualitatively consistent in that CoVPF exhibited higher performance than the other models (S5 Fig).

### New mutations temporally affect forecasting

**Fig 3:**
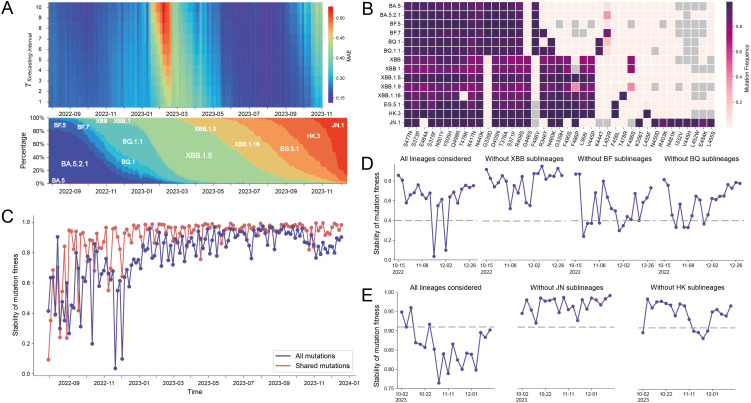
New mutations temporally affected forecasting performance of CoVPF. **(A)** The mean absolute error (MAE) of global forecasts for each 4-day interval increment. *T*_*forecasting interval*_, the rank of 4-day interval in a forecast, with ten 4-day intervals. The emergence and prevalence of the major Omicron lineages are correspondingly shown below. **(B)** Mutation spectrum in the S protein of the lineages shown in A. Mutations that did not appear in lineages are indicated by grey. **(C)** Stability of mutation fitness during August 2022 to December 2023. The stability of mutation fitness at a given time *t* was defined as the Pearson correlation coefficient (PCC) of the mutation fitness estimated at *t* and *t* + 40 days. **(D-E)** The effects of excluding specific lineages on the stability of mutation fitness by excluding specific lineages. The ablation studies were respectively conducted for the periods of October 15, 2022 to December 30, 2022 (D) and October 2, 2023 to December 27, 2023 **(E)**, corresponding to the two drops in the forecasting performance shown in A. The ablated lineages were denoted at the upper of graphs.

Next, we investigated the temporal decrease of CoVPF forecast accuracy (Fig 2A-C). We repeated forecasts in four-day (rather than 40-day) intervals, and plotted the MAEs of all forecasts along with the incidence and prevalence of main lineages (Fig 3A). We found that reduced forecast accuracy coincided with the emergence of XBB.1.5 and JN.1. Notably, both XBB and JN.1 variants were abundant with novel RBD mutations compared to those of their predecessors (Fig 3B and S6 Fig). For example, compared to its predecessor BQ.1.1 which had 19 characteristic RBD mutations, XBB.1.5 discarded three mutations (G339D, F486V and K444T) and exhibited five novel mutations. Similarly, compared to its predecessor HK.3 (24 mutations in RBD), JN.1 discarded seven mutations and gained nine novel mutations.

We thus investigated whether newly emerging lineages with large genomic changes could affect forecasting accuracy. Mutation fitness values estimated by CoVPF are essential parameters for forecasting. Fitness was continuously updated over time as more training data were included. We expected and observed that the values of mutation fitness became increasingly stable with time, as shown in Fig 3C and S7-S9 Figs. Before January 2023, mutation fitness fluctuated substantially, and especially in December 2022 when the largest variation occurred, which might be ascribed to the novel mutations of the proliferating XBB lineages (purple dotted line in Fig 3C). A similar reduction in fitness stability occurred when JN.1 emerged, but to a much smaller extent. We also evaluated the fitness of the twelve mutations shared among lineages from August 2022 to January 2024, and found that stability still dropped in December 2022 when XBB emerged (red dotted line in Fig 3C), indicating mutational epistasis.

We conducted an *in silico* ablation study that demonstrated the effects of XBB and JN.1 on fitness estimates. The large drop in fitness stability was mitigated to a greater degree by omitting XBB rather than BF or BQ-sub-lineages (Fig 3D). Similarly, mutation fitness stability was higher after the exclusion of JN.1 than HK lineages (Fig 3E).

### Forecasting requires regular genomic surveillance

**Fig 4:**
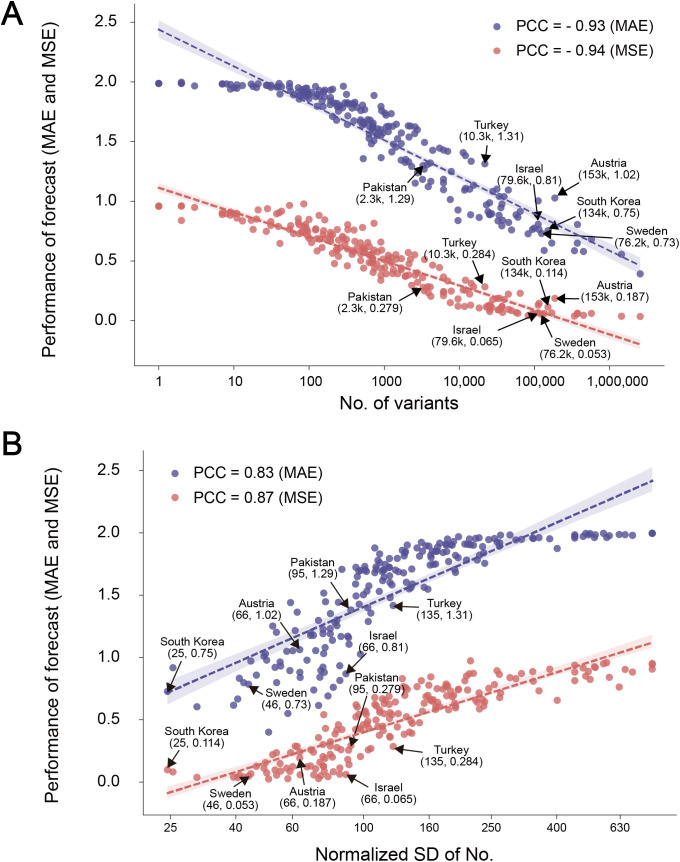
Impact of the amount and regularity of regional sample submissions on forecasts. **(A)** Correlation between sample submission and forecast performance qualified by mean absolute error (MAE) and mean squared error (MSE) for countries or regions. Every country with comparable submission numbers but differed in forecasting performance (see context) with indicated by arrows, and their submission numbers and MAEs or MSEs are marked in the brackets. **(B)** Correlation between normalized standard deviation (SD) of sample submission and forecast performance qualified by MAE and MSE for countries or regions. The normalized SD is defined as the SD when the total submission was normalized to 10,000 for a given country or region. See S16 Fig for the submissions along with time for the labeled countries.

Regional forecasts were based on the training of model with prevalence spectra (*Y*_*T.L*_) for individual regions of the globe. As shown in S5 Fig in the above section, we observed a remarkably higher performance in forecasting for countries with large genomic surveillance datasets such as the USA (*n* = 1.5 million) and the UK (*n* = 350 K) than those reporting relatively few data (e.g., Brazil, 66 K, Poland, 11 K). Therefore, to investigate how scale or *Y*_*T.L*_ affected forecasting, we compared the performance of CoVPF in 195 countries and regions, similar to the global forecast shown in Fig 2A. MAEs and MSEs correlated significantly with the logistic total numbers of variants under genomic monitoring (linear regression, slope < –0.93, *P* < 4 × 10^-96^, Fig 4A). MAEs and MSEs decreased when numbers of regional variants were approximately >400, indicating a baseline of genomic surveillance for forecasting.

Notably, forecast accuracy differed greatly among several countries and regions with comparable numbers of variants. For example, Austria and South Korea were highly similar in data quantity; however, Austria had a 37.3% larger MAE (1.03) and a 64.2% larger MSE (0.187) than South Korea (MAE 0.75, MSE 0.114, Fig 4A). A comparison of Israel and Sweden disclosed a similar finding (Fig 4A). In contrast, variants under genomic surveillance were 4.3-fold higher in Turkey than in Pakistan, but forecasts for these two countries yielded highly similar MAEs and MSEs (Fig 4A).

We thus examined the temporal genomic surveillance in six countries, and found that South Korea, Sweden, and Pakistan sampled more regular and reported to GISAID with time than their counterparts Austria, Israel, and Turkey, respectively (S16 Fig). We used standard deviations (SDs) of a temporal submission normalized to 10 K, which eliminated the effect of different data scales, as a metric of surveillance regularity. In the 195 countries and regions, forecasting performance correlated significantly with SDs (linear regression, slope >0.83, *P* < 2 × 10^-51^), and was positively correlated with SDs smaller than 160 (Fig 4B).

### CoVPF case studies

CoVPF forecasts for the globe and 20 countries and regions that continue to submit data to GISAID are publicly available via http://covpf.com/. The website is updated every few weeks and provides historical forecasts compared with real prevalence from May 14, 2022. Several of CoVPF case studies are presented below.

**Fig 5:**
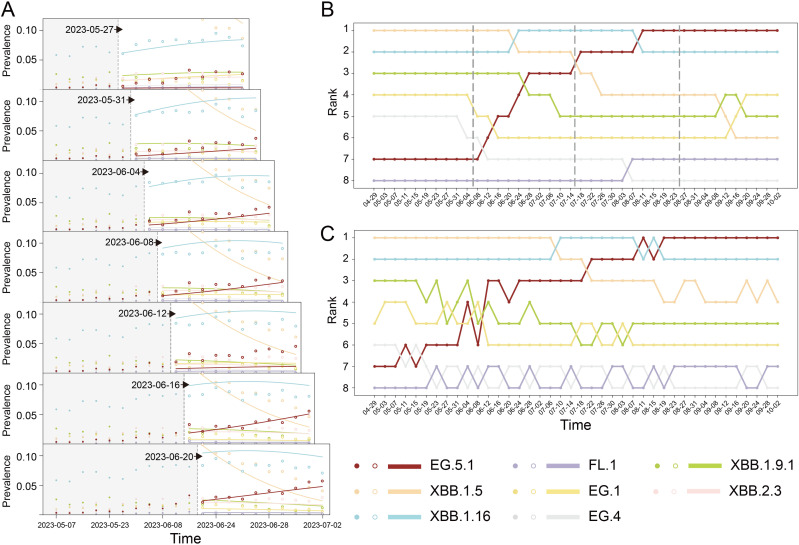
Forecasts of expansion of EG.5.1 in the USA. **(A)** Prevalence forecasts for next 40 days (10 time-intervals) were made every 4 days (1 time-interval) from May 27, 2023 in the USA. The solid dots and hollow dots are the true prevalence of the training set and the validation set respectively. The forecasts of CoVPF are denoted by solid lines. The start dates of forecasts are marked with dotted lines and arrows. **(B-C)** Rank changes in prevalences of lineages for **(B)** CoVPF and **(C)** real in four consecutive forecasts (40 days per forecast from the start date denoted by vertical dashed lines) from 29 April to 2 October 2023.

***Forecasting proliferation of unprecedented EG.5.1 with abundant surveillance.*** The EG.5.1 lineage, a descendant of XBB.1.9.1, was initially detected in the USA in February 2023 *(36)*. At the end of May 2023, XBB.1.5 and XBB.1.16 were dominant (prevalence >7.6%), while EG.5.1 and five other lineages (EG.1, EG.4, FL.1, XBB.1.9.1 and XBB.2.3) circulated at low prevalence rates (<1.5%). On May 31, 2023, the prevalence of EG.5.1 was 1.09%, ranked sixth among the eight circulating lineages; however, CoVPF forecasted that EG.5.1 would increase substantially over the next 40 days, while other lineages were predicted to maintain their low prevalence (Fig 5A). Subsequently, CoVPF correctly forecasted the proliferation of EG.5.1 in the next five four-day forecasts (except on June 12). For comparison, EG.5.1 expansion was forecasted by PyR0 and CM_ranDMS_ on June 4 and by CM_noDMS_ and CM_noEpis_ on June 8, later than CoVPF. Moreover, the other models exhibited higher perturbations than CoVPF in subsequent EG.5.1 forecasts. Performance of forecasts of all models are shown in S10 Fig. We further forecasted the rise of EG.5.1 ranking from April 29th to October 2nd (Fig 5B), which was qualitatively coincident with real temporal rankings (Fig 5C).

**Fig 6:**
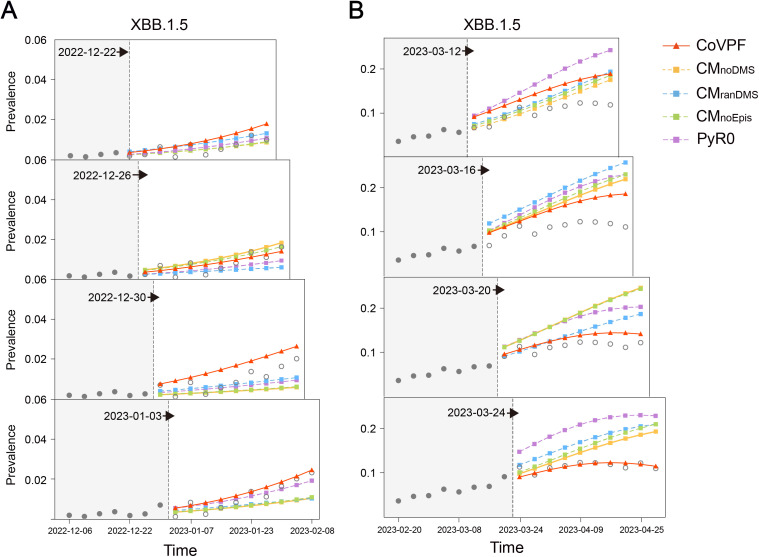
Forecasts of expansion and inflection of XBB.1.5 in South Korea. **(A)** Prevalence forecasts for next 40 days (10 time-intervals) were made every 4 days (1 time-interval) from December 26, 2022. The figure shows the expansion forecasts of CoVPF and other models for the XBB.1.5 in South Korea. The solid dots are the true prevalence of the training set, the hollow dots are the true prevalence of the validation set, and the solid lines are the different model forecasts for XBB.1.5. **(B)** Prevalence forecasts for next 40 days (10 time-intervals) were made every 4 days (1 time-interval) from March 12, 2023. The figure shows the inflection forecasts of CoVPF and other models for the XBB.1.5 in South Korea.

***Forecasting of XBB.1.5 abundant with novel mutations in countries with sufficient and regular surveillance.*** We conducted regional forecasts for the USA, the UK, South Korea, and Austria, all reporting sufficient (*n* > 8000 per month) and regular (SD < 65) genomic data. Because CoVPF forecasting was impeded by newly emerged lineages with abundant novel RBD mutations, such as XBB and JN.1, CoVPF could not deliver a timely forecast of XBB.1.5 proliferation in the USA, where XBB.1.5 variants were first detected (S11A Fig). Surprisingly, in three other countries that experienced delayed introductions of XBB.1.5 (UK, ~ half month; South Korea, ~ one month; Austria, ~ one month later), forecasts of the rise of XBB.1.5 were much more timely than that for the USA (South Korea in Fig 6A and others in S11B-C Figs). Similarly, regional forecasting of the inflection points of XBB.1.5 circulation performed better for the UK, South Korea, and Austria than for the USA (Fig 6B and S11D-F Figs). The CoVPF performance of XBB.1.5 prevalence forecasting surpassed those of the other models (Fig 6 and S11 Fig). For example, CoVPF forecasted the continuous rise of XBB.1.5 in South Korea on December 30, 2022, whereas the forecasts by the other models fluctuated (Fig 6A). The advantage of CoVPF was more obvious when forecasting the inflection of XBB.1.5 in South Korea. On March 12, 2023, CoVPF suggested that an XBB.1.5 inflection would occur after a month, which transpired on April 10, 2023 (Fig 6B). In contrast, the other models failed to forecast the inflection before April 2023.

**Fig 7:**
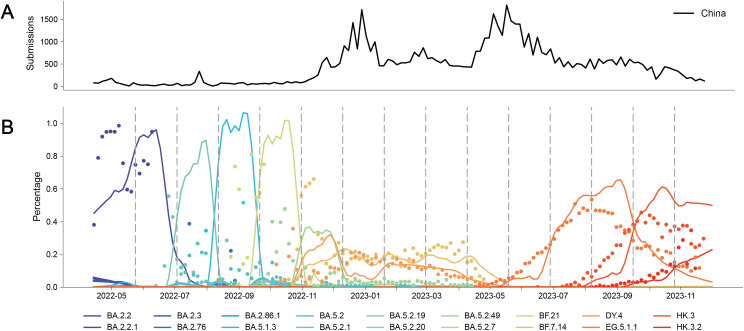
Forecasts in mainland China. **(A)** Variations in sampling submissions every four days of mainland China from April 2022 to December 2023. **(B)** Prevalence forecasts for dominant variants (max prevalence > 0.3) in mainland China. One-time forecast yielded the predicted lineage prevalence at 10 time-intervals (the next 40 days, with a 4-day interval). The time points when the forecasts were made are denoted by vertical dashed lines.

***Forecasting with temporally irregular data.*** Before November 2022, mainland Chinese submissions to GISAID were limited (*n* = 360 ± 110 per month), but then increased rapidly (*n* > 4000 per month, Fig 7A). From April 2022 to November 2023 (1.5 years), we used CoVPF to forecast prevalent lineages in every 40-day interval (Fig 7B). Forecasts made before November 2022 were inaccurate (MAE, 1.31; MSE, 0.497), probably due to limited GISAID data input. We subsequently focused primarily on the forecasts completed after increased data were available. Although forecasting accuracy was improved after November (MAE, 0.75; MSE, 0.075), timeliness was apparently lagging, such as for prevalence predictions of EG.5.1.1 and HK.3. These results implied that irregular data input impeded forecasting.

## Discussion

We developed CoVPF, a tool for forecasting the prevalence of SARS-CoV-2 Omicron lineages. Via a Bayesian multinomial logistic regression model, CoVPF utilizes and combines large-scale genomic surveillance and *in vitro* functional data for practical public health applications, such as forecasting ensuing dominant lineages and the inflection of circulating lineages.

Compared to other methods using genomic epidemiological data, CoVPF’s higher performance demonstrated the benefits of including functional data and accounting for epistasis in forecasting models, making it a strong extension of PyR0 in previous research [30], as it improves forecasting performance while reducing genomic complexity (considering only hundreds of amino acid substitutions within the RBD instead of thousands across the entire genome, as done in PyR0). Control models that disregarded either DMS data (CM_noDMS_) or epistasis (CM_noEpis_) exhibited large performance decrements; whereas the performance of a control model taking epistasis into account but using random DMS values (CM_ranDMS_) was closest to that of CoVPF. This pattern replicated in two additional independently randomized controls, with precise DMS values adding a modest, largely early benefit. Epistasis was embedded by continuously training MLPs in CoVPF, whereas CM_noEpis_ used a linear layer to replace the MLPs. Subsequently, the functional spectra of S-ACE2 affinity (*X*_*LA*_) and immune escape (*X*_*LE*_) could be continuously trained with expanding genomic epidemiological data (*Y*_*T.L*_). Qualitative t-SNE maps place affinity and early escape sites in proximity (S20-S21 Figs), consistent with some epistasis being captured. All results suggest that CoVPF’s forecast performance is driven by the functional modules together with epistasis-capable MLP that learns combinatorial patterns across sites. And even when DMS data were unavailable, the inclusion of artificial functional data and epistasis benefited model construction and training.

CoVPF was used to forecast lineage prevalence in specific countries and regions, in which local (rather than global) epidemiologic data determined *Y*_*T.L*_ for model training. In regional forecasting, the mutation spectrum (*X*_*LM*_) and functional spectra (ACE2 affinity, immune escape) were learned on global data and used as shared inputs across regions. Newly detected lineages were incorporated into all regional forecasts from their first appearance; for regions without observations, the global early-detecting mapping provided an initial regional fitness estimate that enabled early onset prediction. As local sequences accumulated, regional fitness was updated to reflect regional data, and these updates in turn refined the globally shared spectra weights. This global-to-local sharing was adopted to relatively address limited genomic surveillance in many settings and, in practice, regional forecasts benefited from this design. For example, because the USA and the UK contributed nearly half of the global data and frequently reported early detections, CoVPF produced improved forecasts in countries where introductions were delayed, such as that of XBB.1.5 in South Korea and Austria.

This study demonstrated the significance of routine and regular genomic surveillance for forecasting. In addition to our forecasts for countries that have large-scale genomic surveillance, such as the USA and the UK, our prediction for Pakistan, which submitted 2,300 variants to GIASID within a 20 month span, achieved an acceptable accuracy (SD, 95). South Korean submissions were highly regular (SD, 25) and based on 134,000 variants reported over 20 months; CoVPF forecasts of XBB.1.5 prevalence were remarkably accurate and timely. Currently, the scale of SARS-CoV-2 genomic monitoring is rapidly declining. For example, monthly submissions from the USA to the GISAID database decreased from ~5000 to ~1500 variants per month between the end of 2023 and May 2023. Because factors such as variable regional rates of population immunity and public health measures may affect forecasting, we could not propose a threshold for the number of variants that should be monitored per month in a specific country or region. Because SARS-CoV-2 is still spreading and evolving in a large scale in the human population [35–37] and imposing mortality, we urge continuous genomic surveillance as well as public availability of genomic epidemiological data, with hundreds-level sampling that spans diverse subpopulations and geographic settings to ensure representative coverage. Such efforts are crucial for assessing the neutralizing efficacy of antibodies against emerging variants with the potential for rapid prevalence and for ensuring timely vaccine updates.

This study has several limitations. First, because all DMS experiments targeted the RBD, mutation spectrum was limited to the RBD; mutations in non-RBD proteins were disregarded. Consequently, the characterization of lineages may have been incomplete, because lineages are defined by genome-wide nucleotide mutations. Nonetheless, the mutation spectrum, including both mutations and within-lineage frequencies, might partially reduce this bias. Second, CoVPF is a genomic epidemiological model, and various factors such as herd immunity, control measures, and other social factors could not be integrated quantitatively. Our solution is based on the hypothesis that all effects were embedded in lineage prevalence (*Y*_*T.L*_); consequently, we used *Y*_*T.L*_ to train and learn the fitness parameter. However, the exploration of a more comprehensive framework to consider additional factors will be worthwhile. Third, the validation of this study was retrospective. Although we continue to update and release forecasts of SARS-CoV-2 Omicron prevalence, the rapid reduction of genomic surveillance efforts and consequent decrease of real-time data might preclude the future validation of CoVPF forecasts. Fourth, our functional DMS inputs are biased toward earlier Omicron backbones, creating potential context mismatch with later lineages. Future work will require DMS measured on circulating variants, time-aligned and standardized to a common scale across datasets. Fifth, our epistasis analysis is exploratory rather than a full epistasis map, and a comprehensive map across different variant backgrounds remains to be established. Finally, several genome-based virus risk predictors focus on identifying high-risk mutations rather than forecasting lineage prevalence [31–34]. Future work could integrate such predictors into CoVPF as additional priors or covariates, thereby increasing early sensitivity to emerging lineages and strengthening robustness in low-surveillance settings.

## Methods

### Public genomic epidemiology data of omicron variants

The genomic data and related epidemiological metadata of a total of 16.3 million SARS-CoV-2 variants were obtained from the GISAID (https://gisaid.org/) on December 25, 2023, including the amino acid substitutions in the spike protein, sampling locations and time, and viral lineages by Pangolin nomenclature. The variants with a “Omicron” tag were selected for further filtering. In detail, the variants with no detailed sampling dates or missing substitutions of S protein were excluded. Some variants with a “Omicron” tag were found to be assigned to non-Omicron lineages, such as B.1.1 and AY sub-lineages, and they were disregarded. Finally, the data of 8.639 million Omicron variants, belonging to 1824 lineages, were extracted for the following analyses.

### Public functional DMS data of RBD in S protein

The DMS data regarding the functional impacts of single substitutions of the RBD (331–531 positions of the S protein) to the S-ACE2 affinity and immune escape were obtained from the studies by Starr et al [18] and Cao et al [19], respectively. The DMS data for S-ACE2 affinity were generated by mutations of the Omicron BA.1 RBD sequences, downloaded on February 18, 2024, via https://github.com/jbloomlab/SARS-CoV-2-RBD_DMS_Omicron/blob/main/results/final_variant_scores/final_variant_scores.csv. The DMS data were generated by measuring the neutralizing activity of the antibody COVOX-222 against wild-type RBD with all single mutations, downloaded on December 26, 2023, via the link https://media.githubusercontent.com/media/jbloomlab/SARS2_RBD_Ab_escape_maps/main/processed_data/escape_data.csv.

### Construction of prevalence spectra

Based on the genomic epidemiological data, the prevalence spectra of Omicron lineages (3-D tensors for all regions), which recorded the varying of variant counts of different Omicron lineages along with time, were constructed. According to the periodic fluctuations of submissions to GISAID (S1C Fig), We selected a four-day time interval for the variant counting. From November 1, 2021 to December 19, 2023 there were 195 time-intervals. A prevalence spectrum was denoted by *Y*_*T.L*_, where *T* referred to the time and *L* referred to the lineages. The prevalence spectra were respectively constructed for 215 countries and regions as well as globe.

### Construction of mutation spectrum

The information of amino acid substitutions in RBD of the S protein of Omicron lineages were integrated into a mutation spectrum. The mutation spectrum was constructed only based on the global data, and was a 2-D tensor respectively corresponding to the lineages and RBD mutations (denoted as *X*_*LM*_). Namely, the mutation spectrum had no time dimension, and was continuously updated with the expanding genomic data for the following training. To decrease the potential impact of low-frequency mutations on reflecting the characteristics of lineages, the amino acid substitutions whose frequencies were <0.33 in all the lineages were disregarded.

### Construction of functional spectra for S-ACE affinity and immune escape

To integrate the DMS datasets, the continuous values for affinity and escape ability were respectively discretized by dividing into 400 equally spaced intervals in the range. Then, two 3-D tensors were constructed respectively for the S-ACE2 affinity and antibody escape ability. The three dimensions of the 3-D tensors included the lineages, the RBD position, and the discretized functional intervals, and the values were the corresponding intra-lineage substitution frequencies. Next, to take the epistasis into account, the three-layer MLPs were employed to embed the dimension of RBD positions and the intra-lineage frequencies values, and subsequently the 3-D tensors were transferred to 2-D tensors. We also tested five-layer MLPs, and found that they did not increase the performance compared to the three-layer MLPS but they increased the computational expanse. The three-layer MLP consisted of an input layer with 200 neurons followed by a ReLU activation function, a hidden layer with 100 neurons, and an output layer containing a single neuron. The parameters of the MLPs were trained based on the prevalence spectrum (see methods of training in the following sections).

### Hierarchical Bayesian framework

The Pyro probabilistic programming framework developed by Uber AI labs [38] was employed for hierarchical Bayesian regression for fitting and forecasting of CoVPF. In the framework, the joint posterior distribution of all latent variables was calculated by using stochastic variational inference to train a reparametrized variational distribution. The Pyro framework was also used in the study by Obermeyer et al. to estimate fitness of SARS-CoV-2 viral lineages [30].

According to fundamental theorem of natural selection [39], the variation of genotype frequencies follows a logistic curve, and the fitness of the genotypes are defined as the growth rate parameters. Here, for CoVPF, the mutation spectra (*X*_*LM*_), S-ACE2 affinity spectrum (*X*_*LA*_), escape spectrum (*X*_*LE*_), prevalence spectra (*Y*_*TL*_, before the time point) were observable, and latent variables were the initial prevalence (denoted by *α*) and fitness (*β*) of lineages, as well as their variances (*δ*). The latent variables followed the prior distribution, which are shown below and in S3 Fig as

*α*_*L*_ *~ Normal*(*0, δ*_*1*_)*, δ*_*1*_ *~ LogNormal*(*0, 2*),

*α*_*PL*_ *~ Normal*(*α*_*L*_*, δ*_*2*_)*, δ*_*2*_ *~ LogNormal*(*0, 2*),

*β*_*M*_ *~ Laplace*(*0, δ*_*3*_)*, δ*_*3*_ *~ LogNormal*(*-4, 2*),

*β*_*A*_ *~ Laplace*(*0, δ*_*4*_)*, δ*_*4*_ *~ LogNormal*(*-4, 2*),

*β*_*E*_ *~ Laplace*(*0, δ*_*5*_)*, δ*_*5*_ *~ LogNormal*(*-4, 2*),

*β*_*PL*_ *~ Normal*(*∑*_*M*_*β*_*M*_ *X*_*LM*_ *+ ∑*_*A*_*β*_*A*_ *X*_*LA*_ *+ ∑*_*E*_*β*_*E*_ *X*_*LE*_*, δ*_*6*_)*, δ*_*6*_ *~ LogNormal*(*-4, 2*),

*Y*_*TPL*_ *~ Multinomial*(*Y*_*TPL*_*, softmax(α*_*PL*_ *+ β*_*PL*_ ×*T)*),

where *α*_*PL*_ indicated the initial relative prevalence of lineage *L* in region *P*, *β*_*PL*_ was the slope and indicated the fitness of lineage *L* in region *P* (*P* can represent the globe (when *P* = 1) or individual regions (when *P* = number of all regions), so we generalized it with *α*_*.L*_, *β*_*.L*_ and *Y*_*T.L*_ in the previous description), and *β*_*M*_ was the linear regression coefficient representing the effect of a single mutation on the fitness of lineages. The varying of lineage proportions with time (*T*) in a given region (*P*) was represented by the multivariate logistic growth function **softmax(*α*_*PL*_* + β*_*PL*_ × *T)*. The SoftMax function was defined as *softmax(x*_*i*_*) = exp*(*x*_*i*_)/ *sum*_*i*_^*n*^(*exp*(*x*_*i*_)), where *x*_*i*_ equaled to *(α*_*PL*_* + β*_*PL*_ × *T)* in CoVPF, *i* was a given lineage *L* and *n* was the number of all lineages.

### CoVPF for fitting

In the scenario of fitting of the varying of lineage prevalence with time, the latent variables of CoVPF model were inferred based the four spectra constructed till December 19, 2023 (included 195 4-day time-intervals). In detail, the MLPs for S-ACE2 affinity spectrum *X*_*LA*_ and immune escape spectrum *X*_*LE*_ were first trained. Then, a Bayesian logistic regression model was employed to fit the regression of *Y*_*TPL*_, *X*_*LM*_, *X*_*LA*_ and *X*_*LE*_. The inferencing of parameters was performed with the Adam optimizer for 8,000 iterations, and the learning rate set at 0.01. Finally, 1000 samples were drawn from the posterior distributions, and the mean values were used as the final estimates.

### CoVPF for forecasting

For forecasting of lineage prevalence with time, we used expanding dataset to forecast the prevalence of lineages for the next 10 time-intervals (40 days) by CoVPF, starting at any time point. We selected 10 time-intervals (40 days) as the optimal up-limit length of CoVPF, considering the trade-off between the accuracy and practical applications.

In a forecast starting at time-interval *t*, the data preceding *t* was employed as the training set to obtain the inputs (values = 0 for lineages and mutations that did not occur), and then trained to fit the regression. Following the training with the Adam optimizer for 8,000 iterations and the learning rate set at 0.01, the parameters *α*_*PL*_ and *β*_*PL*_ of the logistic growth function were obtained by the mean values of 1000 sample. The proportion of lineage *L* in region *P* over the next 40 days of *t* (with 10 4-day intervals) were *softmax*(*α*_*PL*_* + β*_*PL*_ × (*t* + *i*))*, i = *1,2,…,10*.* CoVPF was continuously trained with an expanding dataset for following forecasts over time.

### Selection of forecast time-interval

In order to clarify the upper limit of forecast, we conducted global forecasts for different time increments (1, 2, 5, 10, 13, 15, 20, and 25 time-intervals, one of which was 4 days), with the first 8 months as initial training datasets (November 2021 to June 2022). Then, the sum of MAE and MSE of forecasts of lineage prevalence from June 2022 to December 25, 2023 were obtained (S15 Fig). We finally chose 10 time-intervals (40 days) as the forecast length in this study.

### Statistical analysis

Mean Absolute Error (MAE) and Mean Squared Error (MSE) in the research were calculated as follows:


$$MAE=\;\frac{\text{1}}{n}\sum_{i=1}^{n}\left|y_{i}-\hat{y_{i}}\right|$$

$$MSE=\;\frac{\text{1}}{n}\sum_{i=1}^{n}({y_{i}-\hat{y_{i}})}^{\text{2}}$$

where *y*_*i*_ represents the observed values and $\hat{y_{i}}$ denotes predicted values.

The Kolmogorov-Smirnov tests were employed to assess the consistency of data distributions in mutation frequencies. And Pearson’s correlation coefficients were calculated to evaluate the strength and direction of the linear relationship between values (forecast accuracy with submission and regularity). Statistical significances were determined using p-value threshold of *p* < 0.05.

Densities of mutation frequency across S protein position intervals was computed using R with the ggplot2 package. Data visualization was performed using Python’s Matplotlib and Seaborn libraries to create informative plots that effectively illustrate the results.

## Supporting information

S1 Fig Summary of genomic surveillance of SARS-CoV-2 and S protein mutations. (A-B) Numbers of variants (A) and lineages (B) of variants of concern (VOCs) in GISAID database till December 19, 2023. (C) Total submissions of SARS-CoV-2 surveillance to GISAID along with time from November 1, 2021 to December 19, 2023. (D) Relative fractions of major lineages from November 1, 2021 to December 19, 2023. (E) Distributions of submissions by the top ranked countries. (F) Shannon diversity index for each position in Omicron spike protein. The structure of spike protein is shown below, and the numbers in bracket are the numbers of positions with a Shannon diversity no less than 0.1. Shannon diversity index is defined as *-∑*_*i*_*(P*_*i*_*lnP*_*i*_), where *P*_*i*_ is the proportion of the number of an amino acid substitution *i* to the number of all amino acid substitutions at the position on S protein. (G) Density of distribution of minor allele frequencies of mutations in NTD and RBD of Omicron variants.
(PDF)

S2 Fig Surveillance of endemic lineages in the USA, the UK, Germany, France, Brazil and Poland. (PDF)

S3 Fig Probabilistic graphical model structure of CoVPF. (PDF)

S4 Fig Evaluation of CoVPF performance and comparison with three control models and PyR0. (A) Fitting of prevalence in the globe until December 2023. CM_noDMS_, the control model that ignored deep mutational scanning (DMS) data. CM_ranDMS_, the control model using random values for DMS. CM_noEpis_, the control model did not consider epistasis. PyR0, the model considered whole genome of SARS-CoV-2 and ignored DMS data. (B-C) The performance of fitting. Mean absolute error (MAE, B) and mean squared error (MSE, C) were used as metrics.
(PDF)

S5 Fig CoVPF performance of prevalence (A) fitting and (B) forecasting for lineages in the USA, UK, Germany, France, Brazil, and Poland. (PDF)

S6 Fig RBD sequence similarity between prevalent lineages in different periods. Except for XBB and its predecessor BQ.1.1, and JN.1 and its predecessor HK.3, which were less similar (< 80%), the similarity between other lineages was high.
(PDF)

S7 Fig Stabilities of mutation fitness at different time intervals. (PDF)

S8 Fig Stabilities of mutation fitness at all time intervals. (PDF)

S9 Fig Fitness of previous lineages before and after the emergence of prevalent lineages. Estimates of CoVPF fitness to previous lineages before and after the emergence of prevalent lineages in different time periods did not change excessively. However, mutation fitness will change more.
(PDF)

S10 Fig Benchmark of forecasting the growth of EG.5.1 in the USA. (A) Changes in percentage ranks in CoVPF forecasts. (B) Changes in percentage ranks in CM_noDMS_ forecasts. (C) Changes in percentage ranks in CM_ranDMS_ forecasts. (D) Changes in percentage ranks in CM_noEpis_ forecasts. (E)Changes in percentage ranks in PyR0 forecast. (F) Forecasts of EG.5.1 rise from five models.
(PDF)

S11 Fig Inflection forecast of XBB.1.5 in regions. (A) Forecast the rise of XBB.1.5 in the USA. (B) Forecast the rise of XBB.1.5 in the UK. (C) Forecast the rise of XBB.1.5 in Austria. (D) Forecast the inflection of XBB.1.5 inflection in the USA. (E) Forecast the inflection of XBB.1.5 inflection in the UK. (F) Forecast the inflection of XBB.1.5 inflection in Austria. The red pentagram means that the CoVPF forecasted the inflection of XBB.1.5 using the data from the cut-off date in the current figure.
(PDF)

S12 Fig Fitness analyses of lineages with > 10,000 or 5,000 submissions globally. (A) Fitness analysis of global Omicron lineages whose submissions > 10,000. The x axis represents the time of emergence, the y axis represents CoVPF fitness estimates, and the scatter size represents the scale of submission. Red scatters are non-recombinant lineages, blue scatters are recombinant lineages. (B) Distribution of the difference between regression values and fitness after regressing non-recombinant and recombinant lineages with submissions > 10,000 using linear regression. (C) Kernel density distributions of non-recombinant and recombinant lineages. Kolmogorov-Smirnov (K-S) test, p = 0.11, non-recombinant and recombinant lineages whose submissions > 1,0000 have the same distribution. Fitness analyses of lineages with > 5,000 submissions globally. (D) Relationship between lineages’ emergence time and fitness whose submissions > 5,000. € Distribution of the difference between regression values and fitness after regressing non-recombinant and recombinant lineages with submissions > 5,000 using linear regression. (F) Kernel density distributions of non-recombinant and recombinant lineages whose submissions > 5,000. Kolmogorov-Smirnov (K-S) test, p = 0.051, non-recombinant and recombinant lineages have the same distribution.
(PDF)

S13 Fig Distribution of lineage fitness occurring in each region over time. (PDF)

S14 Fig Correlation tests of fitness of co-occurring lineages across different regions. (PDF)

S15 Fig Performance of global forecast for different time increments (1, 2, 5, 10, 13, 15, 20, and 25 time-intervals, one of which was 4 days). (PDF)

S16 Fig Variations in sampling submissions every four days of (A) Austria, (B) South Korea, (C) Israel, (D) Sweden, (E) Turkey, and (F) Pakistan from April 2022 to December 2023. (PDF)

S17 Fig Fitness analysis of Omicron lineages and RBD mutations. (A) Fitness analysis of global Omicron lineages. The *x* axis represents the date at which the variant was first observed in GISAID, the y axis represents the relative fitness of lineages estimated by CoVPF, and the scatter size represents the scale of submission. The relative fitness of lineage was calculated by dividing the fitness of lineage estimated by CoVPF by the fitness of B.1.1.529 estimated by CoVPF. Red scatters are non-recombinant lineages, and blue scatters are recombinant lineages. (B-E) Fitness analyses of Omicron lineages in (B) the USA; (C) the UK; (D) South Korea; (E) mainland China. (F) Fitness analysis of RBD mutations. Grey squares represent no mutation information.
(PDF)

S18 Fig The performance of forecasting with time. (A) Mean absolute errors (MAEs) and (B) mean squared errors (MSEs) were used to quantified the performance of forecasts corresponding to CoVPF, CMranDMS_ver1 (original CMranDMS), CMranDMS_ver2 and CMranDMS_ver3.
(PDF)

S19 Fig MLP embeddings visualisation. (A) First-layer MLP weights across models. Heatmaps of the first-layer weight matrices (fc1; 100 × 200) for CoVPF and the three independently randomized-DMS controls (CMranDMS_ver1-ver3). (B) Pairwise Pearson correlation coefficients between the first-layer MLP weight matrices for CoVPF and CMranDMS_ver1-ver3.
(PDF)

S20 Fig t-SNE of learned site embeddings from the affinity MLP. First-layer weights (fc1; columns = 200 RBD sites) are projected into 2D; points represent sites. Functionally representative affinity-related sites for this analysis are highlighted in red with labels.
(PDF)

S21 Fig t-SNE of learned site embeddings from the escape MLP (training truncated at Aug 2022). First layer weights are projected into 2D as in Fig 3. Escape-related sites for the Aug 2022 period are highlighted in red with residue labels.
(PDF)

S1 Table Rank of mutation fitness. (XLSX)

S2 Table Key modeling details for CoVPF and controls. (XLSX)
