# Supplementary figures and images for: Integrating genomic epidemiology and deep mutational scanning data for prevalence forecasting of SARS-CoV-2 Omicron lineages
Source: PLoS One. 2025 Nov 3;20(11):e0335520. doi: 10.1371/journal.pone.0335520 (PMC12582474; doi:10.1371/journal.pone.0335520)

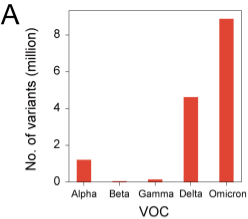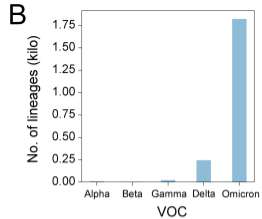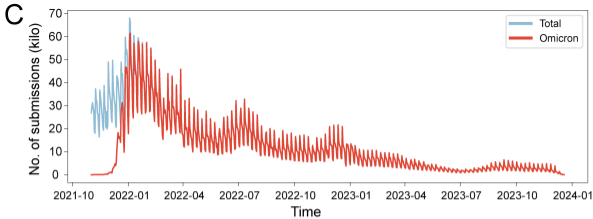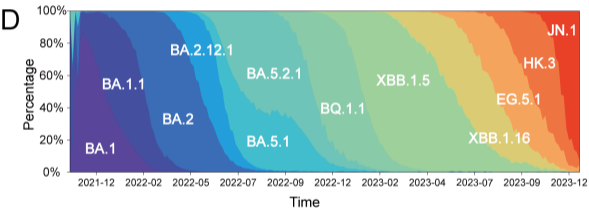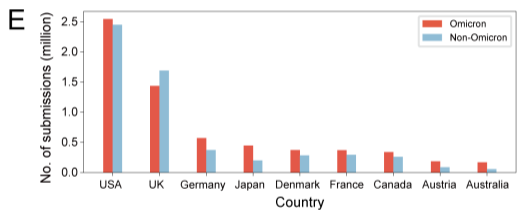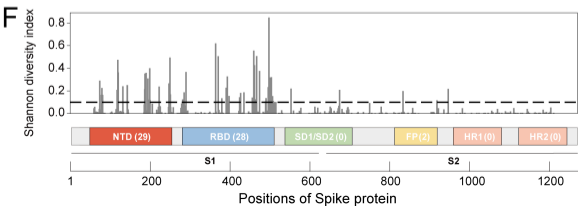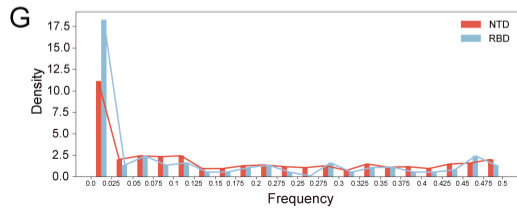

Supplement: S1 Fig — (A-B) Numbers of variants (A) and lineages (B) of variants of concern (VOCs) in GISAID database till December 19, 2023. (C) Total submissions of SARS-CoV-2 surveillance to GISAID along with time from November 1, 2021 to December 19, 2023. (D) Relative fractions of major lineages from November 1, 2021 to December 19, 2023. (E) Distributions of submissions by the top ranked countries. (F) Shannon diversity index for each position in Omicron spike protein. The structure of spike protein is shown below, and the numbers in bracket are the numbers of positions with a Shannon diversity no less than 0.1. Shannon diversity index is defined as -∑i(PilnPi), where Pi is the proportion of the number of an amino acid substitution i to the number of all amino acid substitutions at the position on S protein. (G) Density of distribution of minor allele frequencies of mutations in NTD and RBD of Omicron variants. (PDF) [file pone.0335520.s001.pdf]

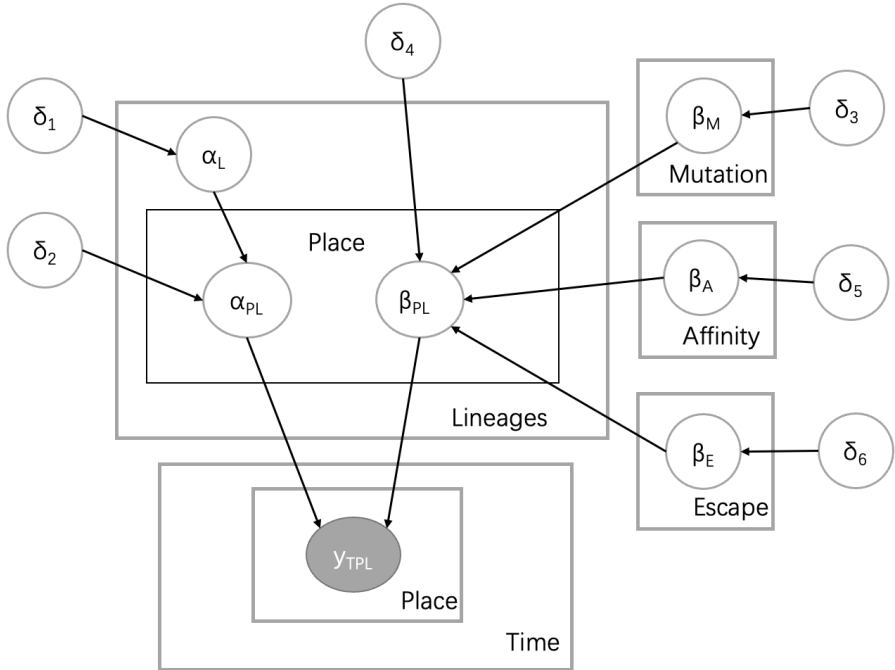

Supplement: S3 Fig — (PDF) [file pone.0335520.s003.pdf]

**A**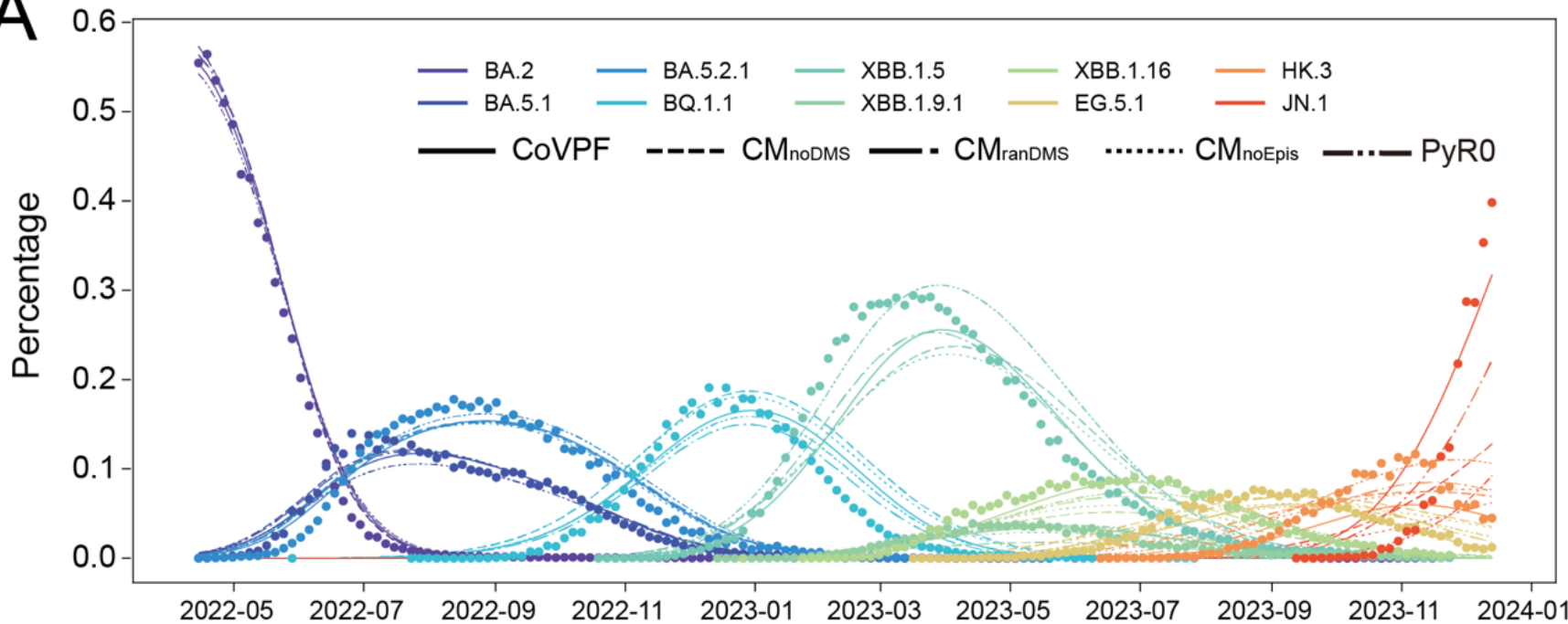**B**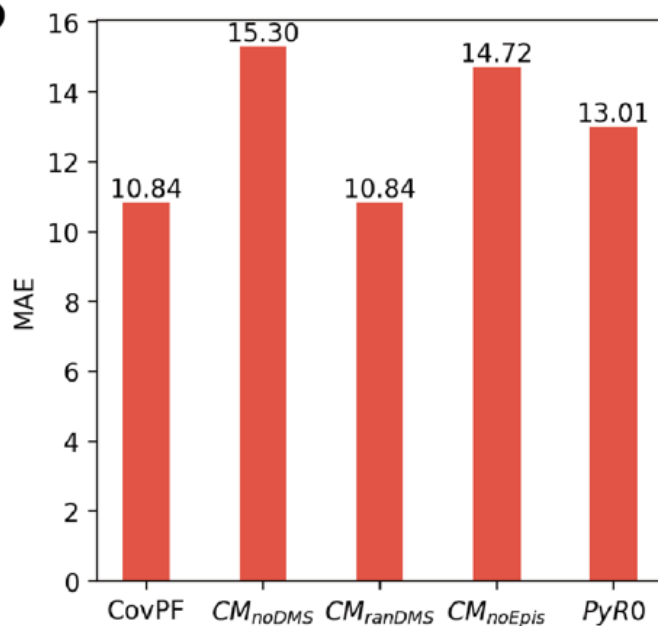**C**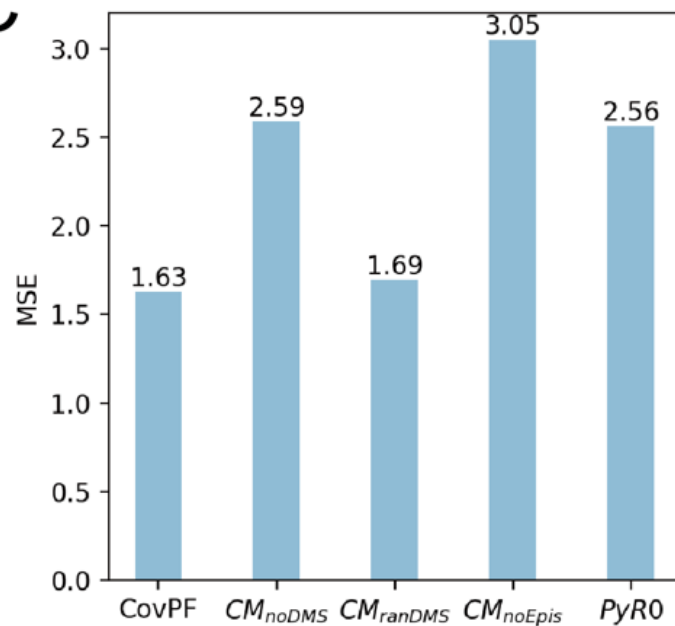

Supplement: S4 Fig — (A) Fitting of prevalence in the globe until December 2023. CMnoDMS, the control model that ignored deep mutational scanning (DMS) data. CMranDMS, the control model using random values for DMS. CMnoEpis, the control model did not consider epistasis. PyR0, the model considered whole genome of SARS-CoV-2 and ignored DMS data. (B-C) The performance of fitting. Mean absolute error (MAE, B) and mean squared error (MSE, C) were used as metrics. (PDF) [file pone.0335520.s004.pdf]

A

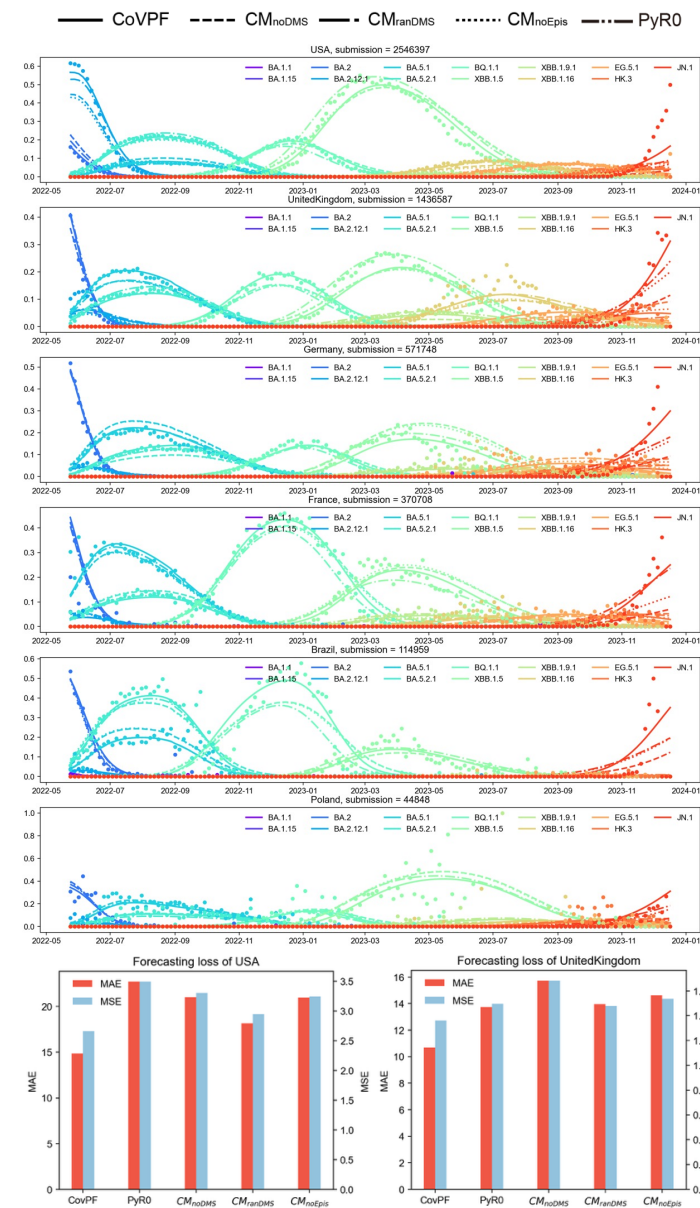

B

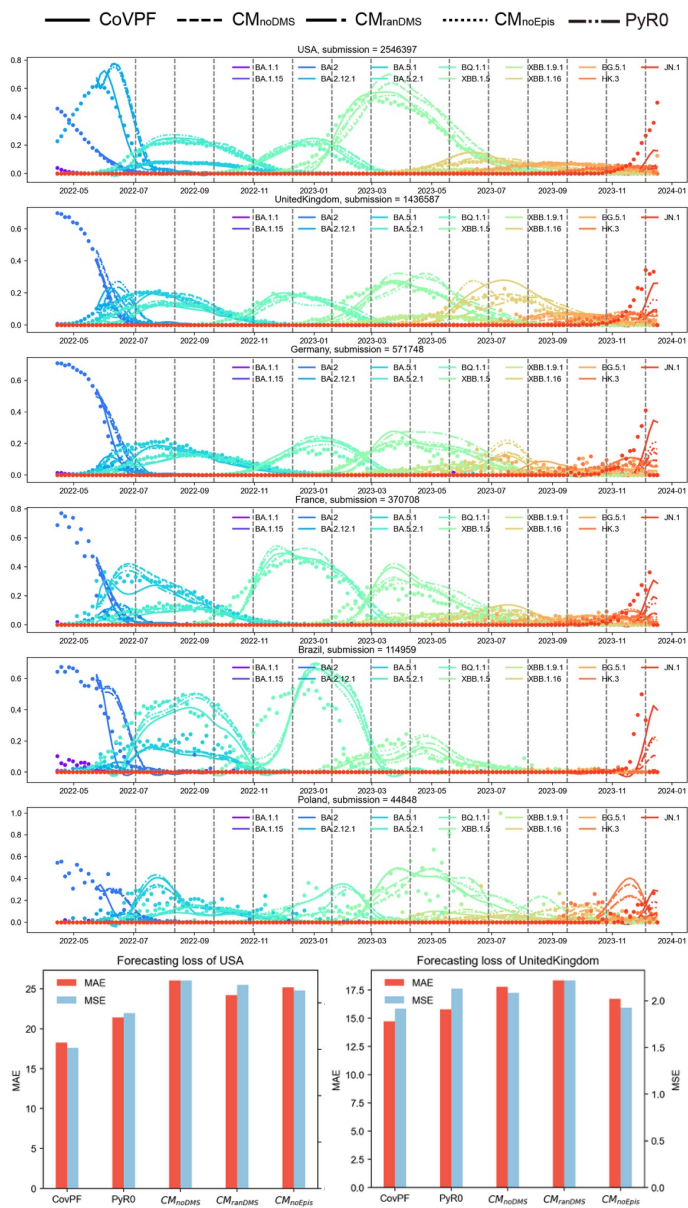

Supplement: S5 Fig — (PDF) [file pone.0335520.s005.pdf]

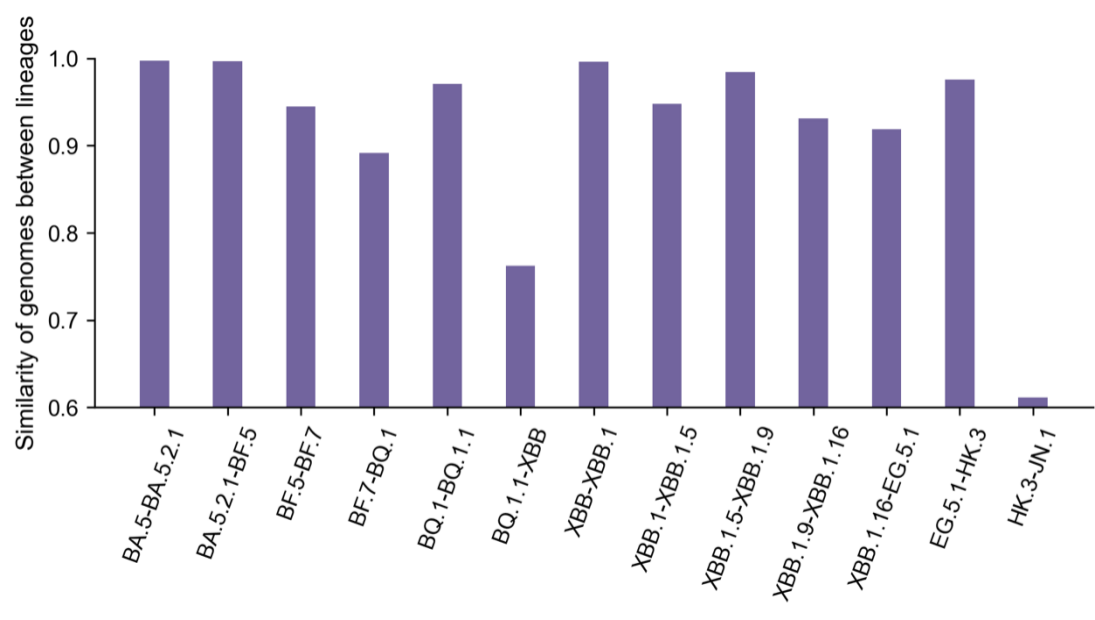

Supplement: S6 Fig — Except for XBB and its predecessor BQ.1.1, and JN.1 and its predecessor HK.3, which were less similar (< 80%), the similarity between other lineages was high. (PDF) [file pone.0335520.s006.pdf]

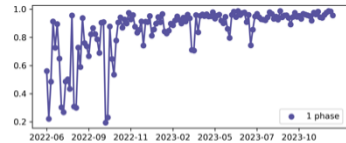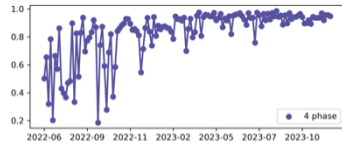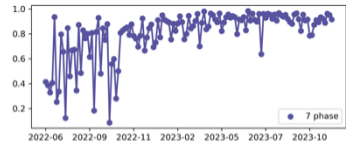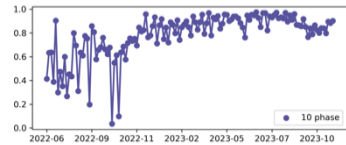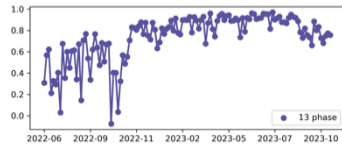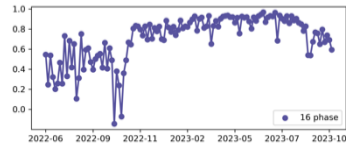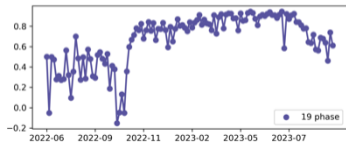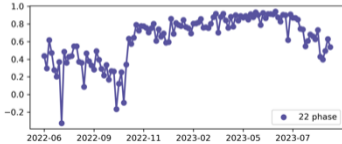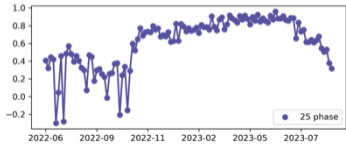

Supplement: S7 Fig — (PDF) [file pone.0335520.s007.pdf]

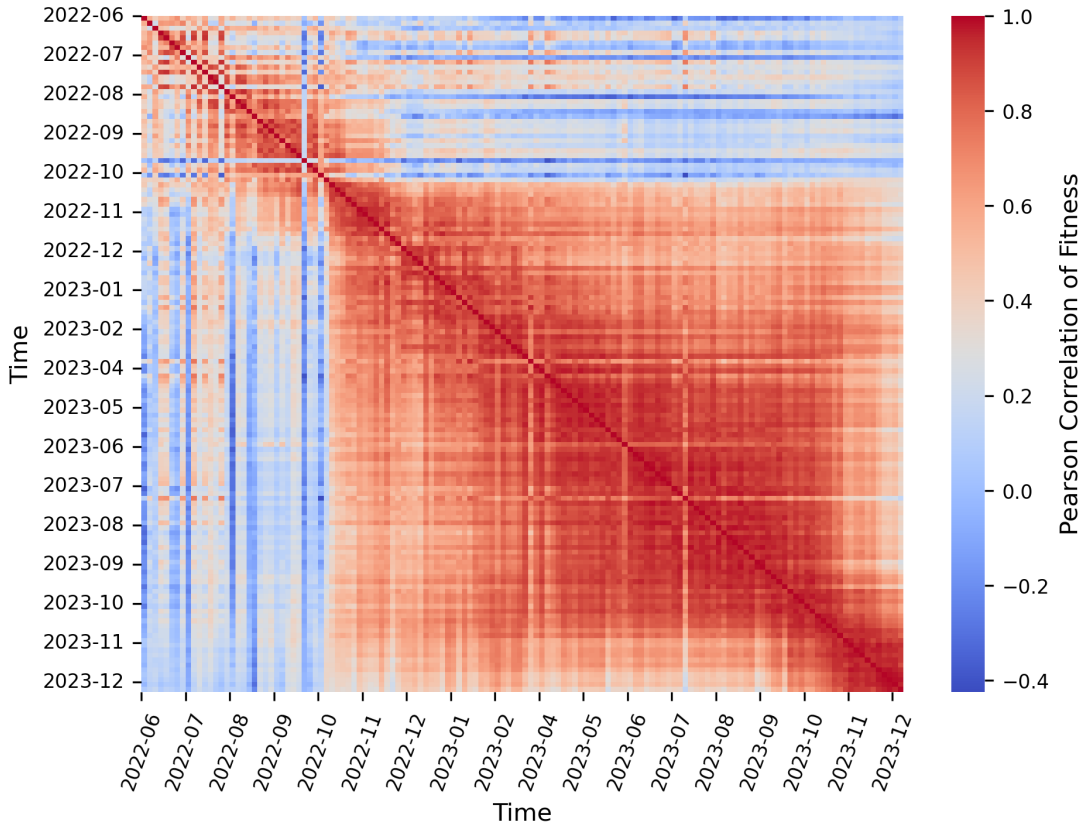

Supplement: S8 Fig — (PDF) [file pone.0335520.s008.pdf]

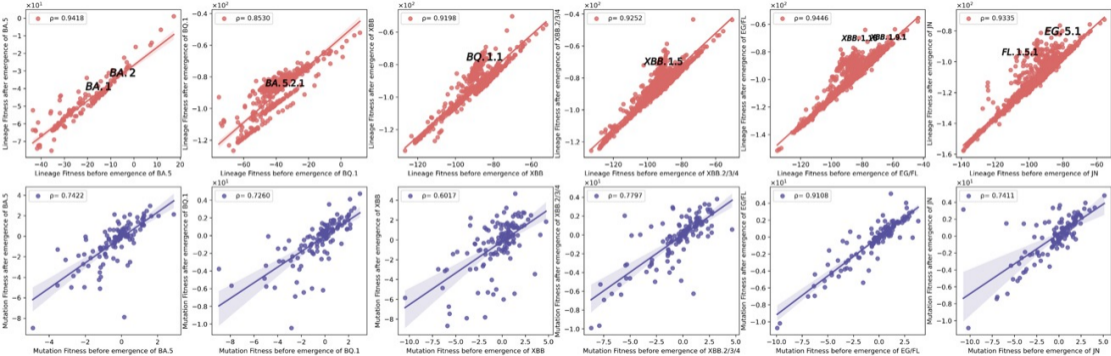

Supplement: S9 Fig — Estimates of CoVPF fitness to previous lineages before and after the emergence of prevalent lineages in different time periods did not change excessively. However, mutation fitness will change more. (PDF) [file pone.0335520.s009.pdf]

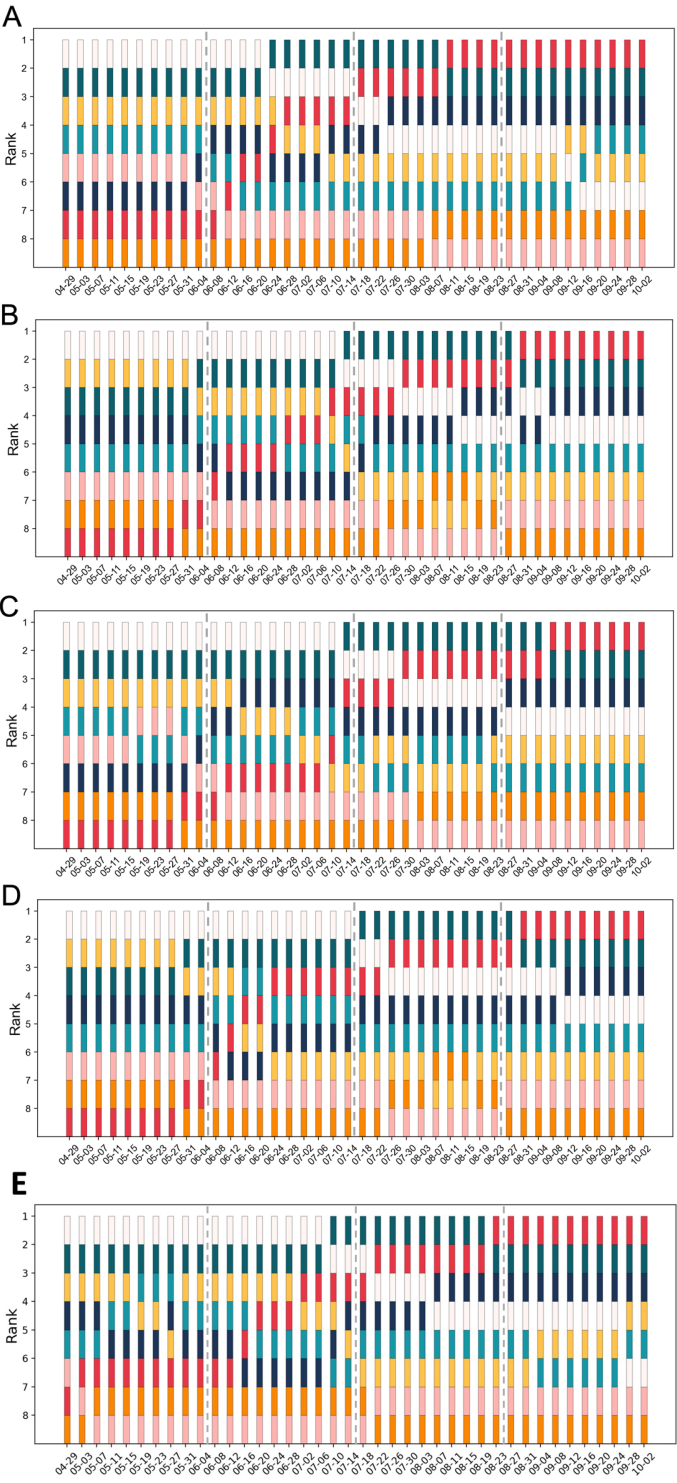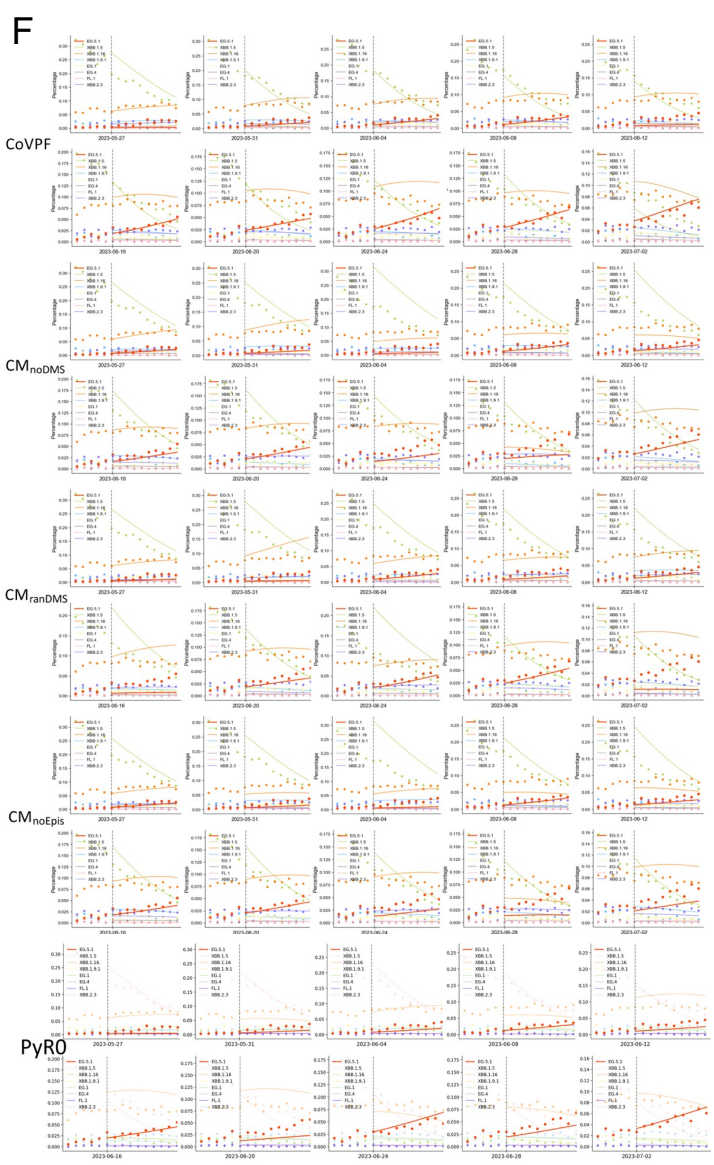

Supplement: S10 Fig — (A) Changes in percentage ranks in CoVPF forecasts. (B) Changes in percentage ranks in CMnoDMS forecasts. (C) Changes in percentage ranks in CMranDMS forecasts. (D) Changes in percentage ranks in CMnoEpis forecasts. (E)Changes in percentage ranks in PyR0 forecast. (F) Forecasts of EG.5.1 rise from five models. (PDF) [file pone.0335520.s010.pdf]

**A**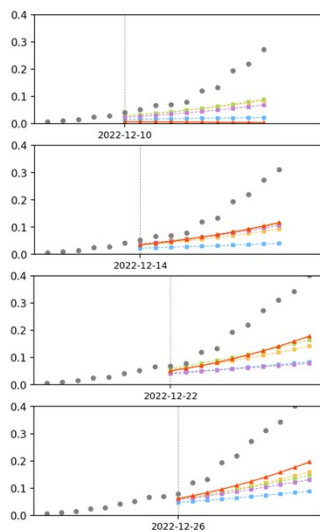**B**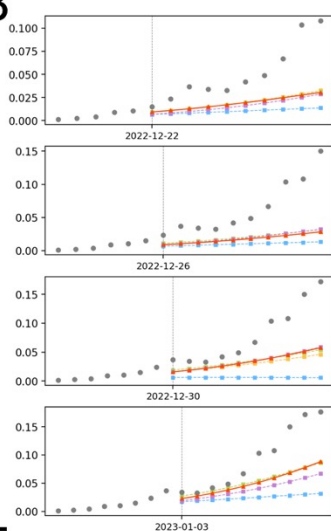**C**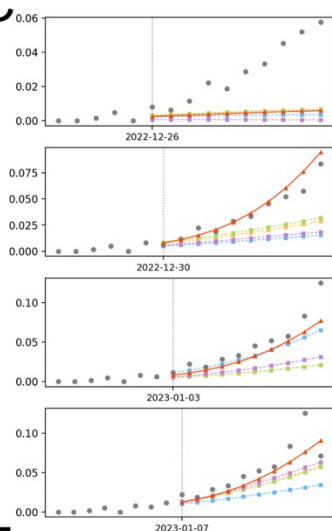**D**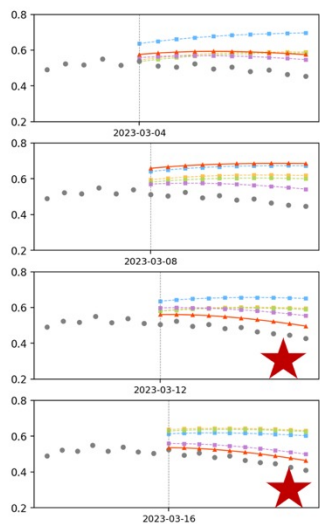**E**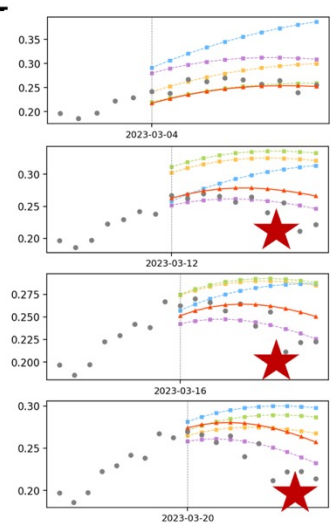**F**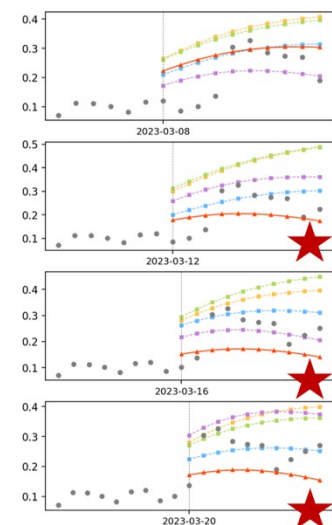

CoVPF

CMnoDMS

CMranDMS

CMnoEpis

PyR0

Supplement: S11 Fig — (A) Forecast the rise of XBB.1.5 in the USA. (B) Forecast the rise of XBB.1.5 in the UK. (C) Forecast the rise of XBB.1.5 in Austria. (D) Forecast the inflection of XBB.1.5 inflection in the USA. (E) Forecast the inflection of XBB.1.5 inflection in the UK. (F) Forecast the inflection of XBB.1.5 inflection in Austria. The red pentagram means that the CoVPF forecasted the inflection of XBB.1.5 using the data from the cut-off date in the current figure. (PDF) [file pone.0335520.s011.pdf]

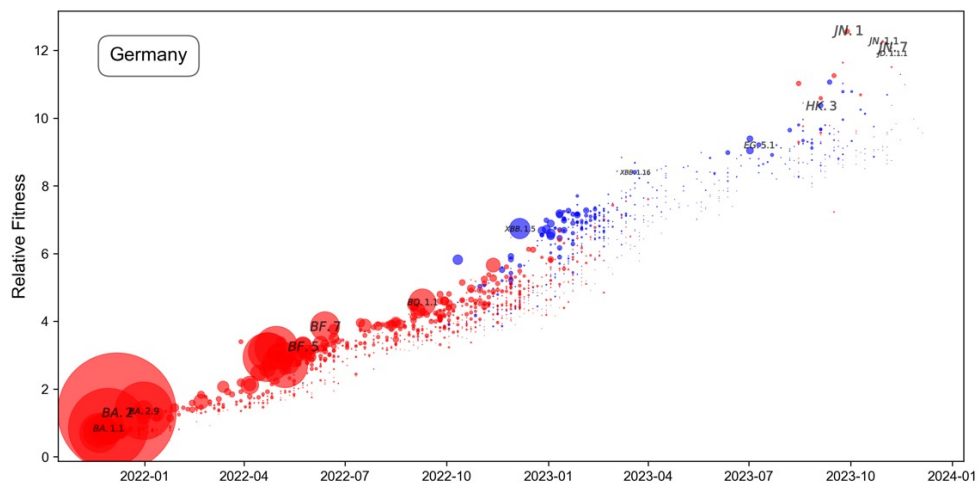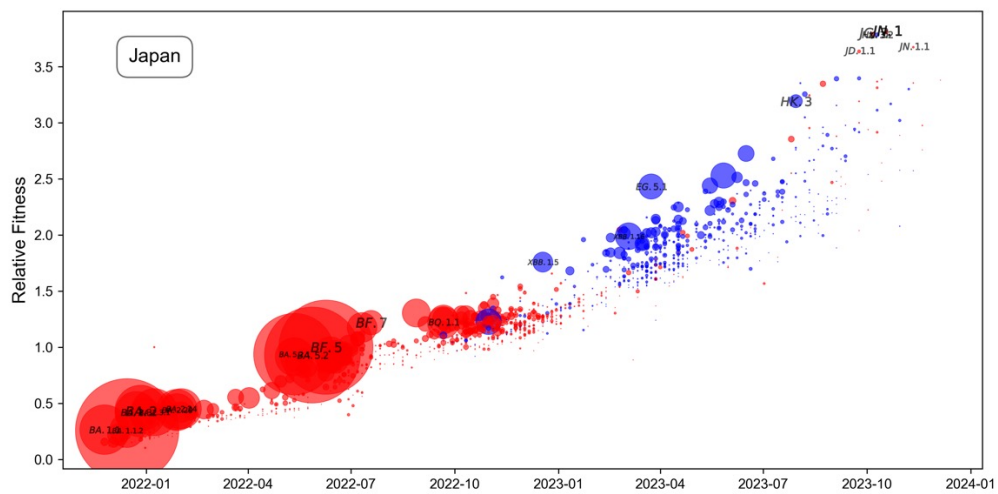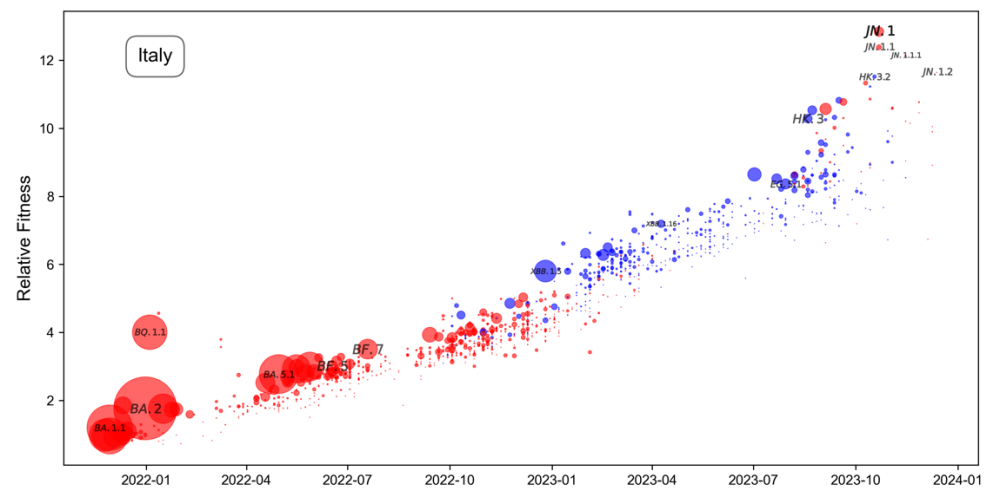

Supplement: S13 Fig — (PDF) [file pone.0335520.s013.pdf]

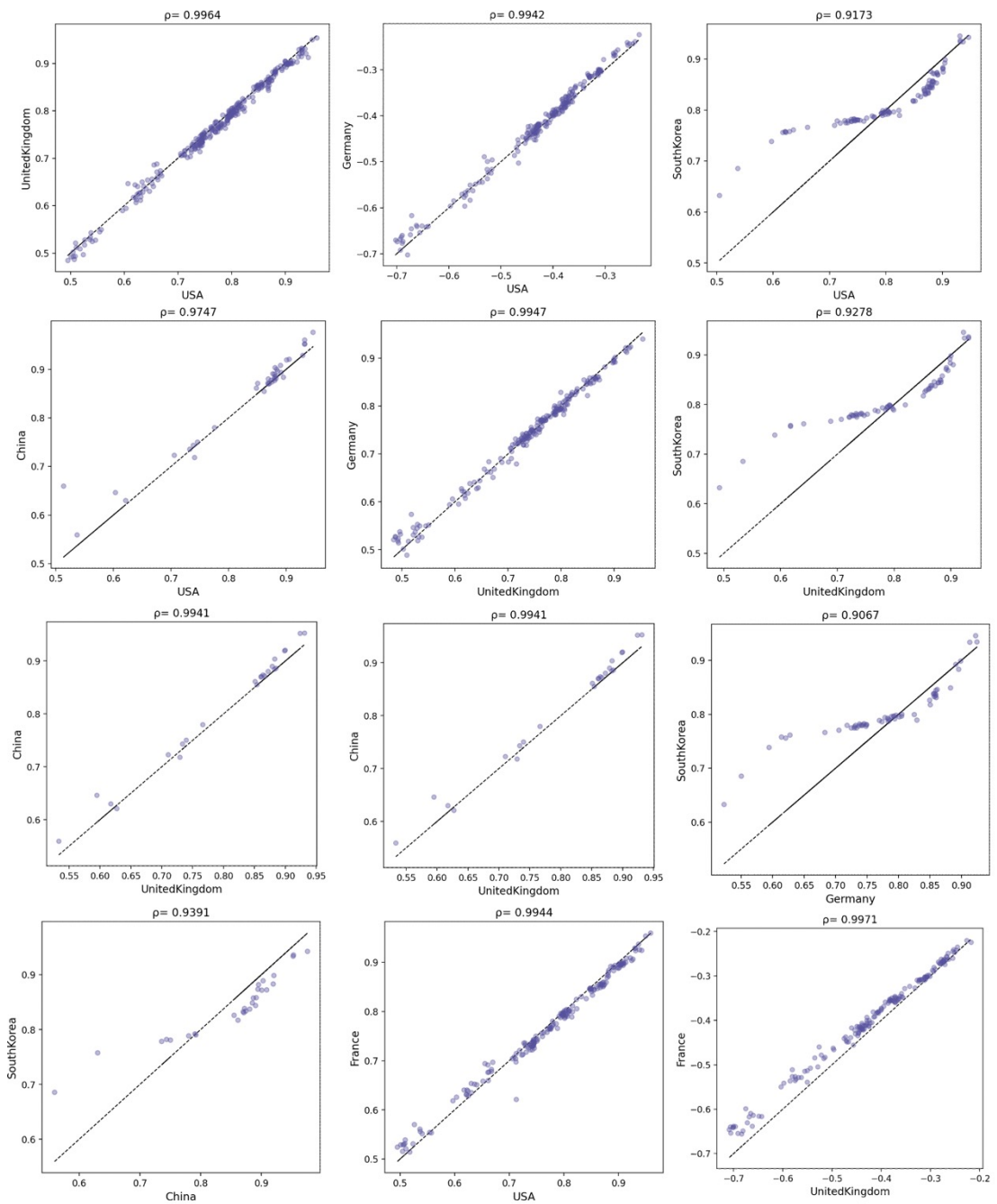

Supplement: S14 Fig — (PDF) [file pone.0335520.s014.pdf]

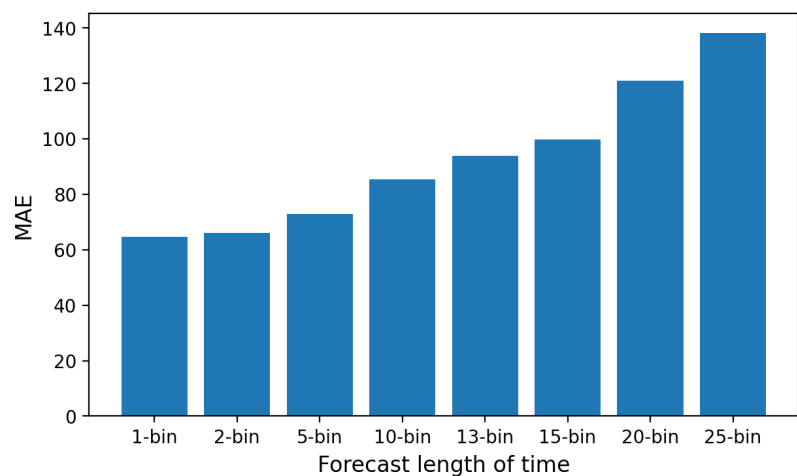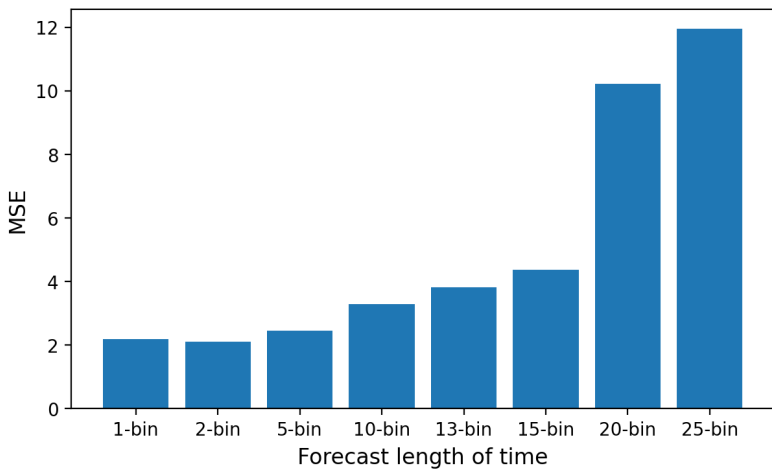

Supplement: S15 Fig — (PDF) [file pone.0335520.s015.pdf]

**A**

Austria

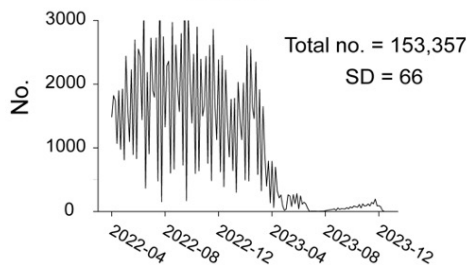**B**

South Korea

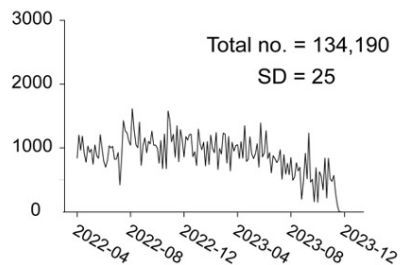**C**

Israel

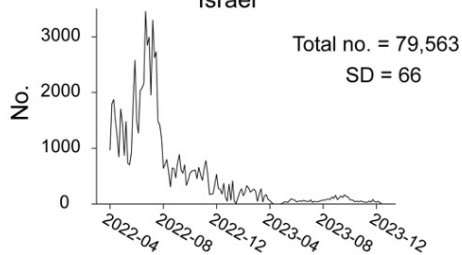**D**

Sweden

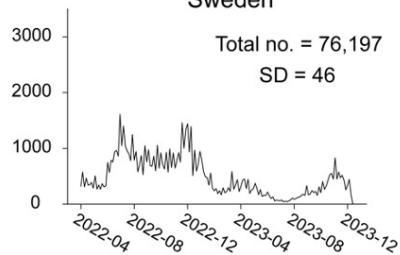**E**

Turkey

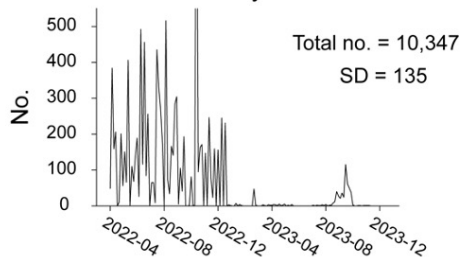**F**

Pakistan

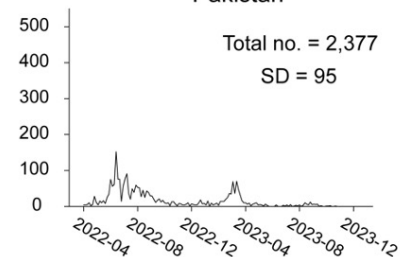

Supplement: S16 Fig — (PDF) [file pone.0335520.s016.pdf]

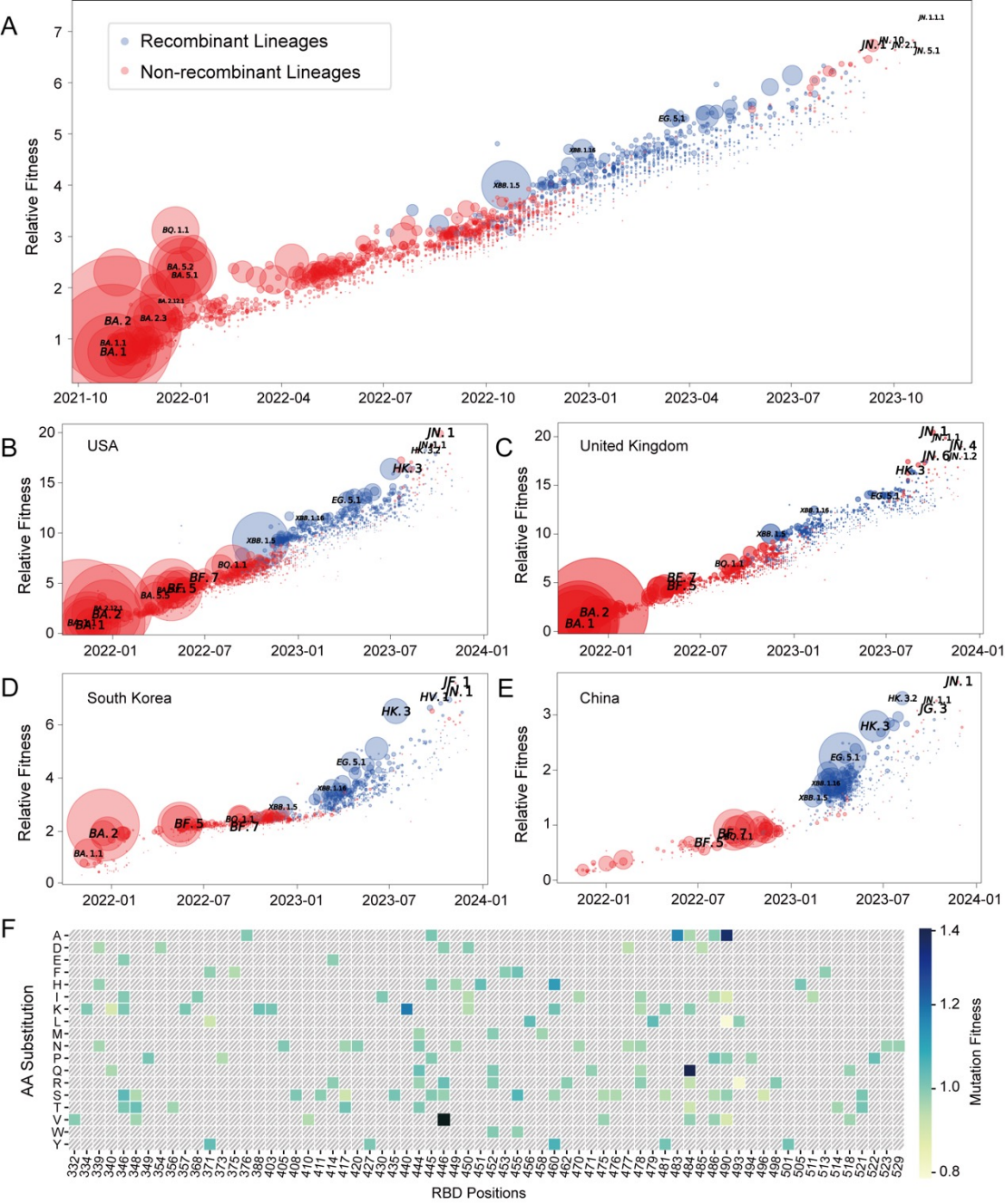

Supplement: S17 Fig — (A) Fitness analysis of global Omicron lineages. The x axis represents the date at which the variant was first observed in GISAID, the y axis represents the relative fitness of lineages estimated by CoVPF, and the scatter size represents the scale of submission. The relative fitness of lineage was calculated by dividing the fitness of lineage estimated by CoVPF by the fitness of B.1.1.529 estimated by CoVPF. Red scatters are non-recombinant lineages, and blue scatters are recombinant lineages. (B-E) Fitness analyses of Omicron lineages in (B) the USA; (C) the UK; (D) South Korea; (E) mainland China. (F) Fitness analysis of RBD mutations. Grey squares represent no mutation information. (PDF) [file pone.0335520.s017.pdf]

A

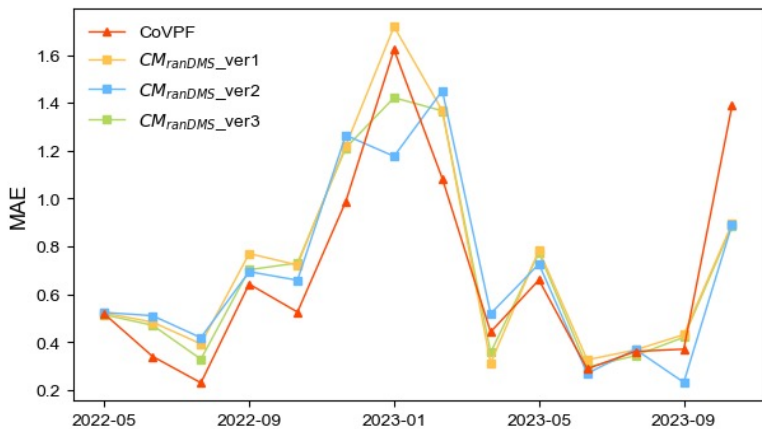

B

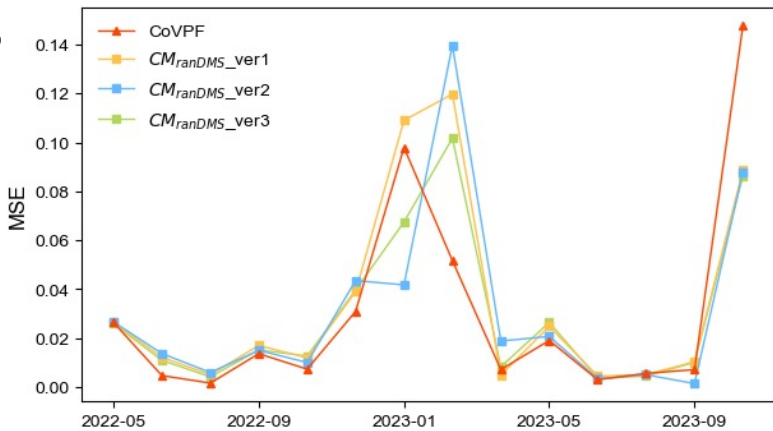

Supplement: S18 Fig — (A) Mean absolute errors (MAEs) and (B) mean squared errors (MSEs) were used to quantified the performance of forecasts corresponding to CoVPF, CMranDMS_ver1 (original CMranDMS), CMranDMS_ver2 and CMranDMS_ver3. (PDF) [file pone.0335520.s018.pdf]

t-SNE

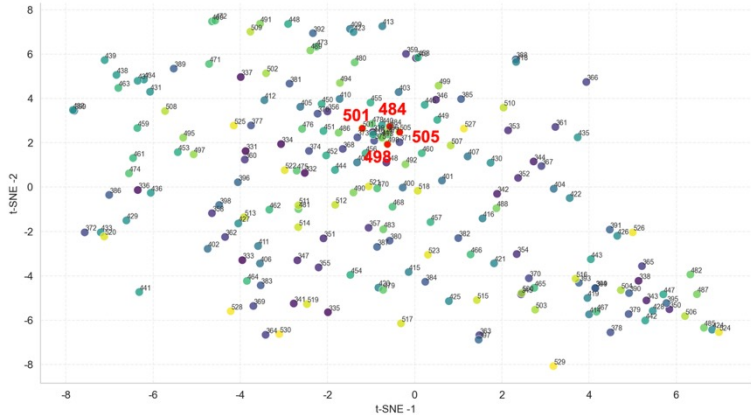

Supplement: S20 Fig — First-layer weights (fc1; columns = 200 RBD sites) are projected into 2D; points represent sites. Functionally representative affinity-related sites for this analysis are highlighted in red with labels. (PDF) [file pone.0335520.s020.pdf]

t-SNE -1

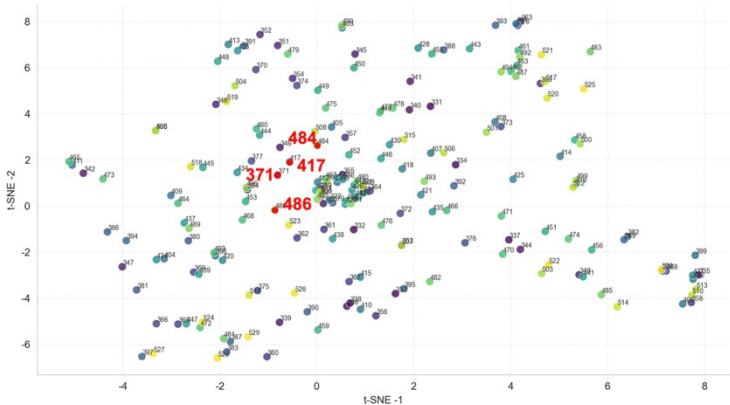

Supplement: S21 Fig — First layer weights are projected into 2D as in Fig 3. Escape-related sites for the Aug 2022 period are highlighted in red with residue labels. (PDF) [file pone.0335520.s021.pdf]
