# Supplementary material for: Integrating genomic epidemiology and deep mutational scanning data for prevalence forecasting of SARS-CoV-2 Omicron lineages
Source: PLoS One. 2025 Nov 3;20(11):e0335520. doi: 10.1371/journal.pone.0335520 (PMC12582474; doi:10.1371/journal.pone.0335520)
Supplement: S2 Fig — (PDF) [file pone.0335520.s002.pdf]

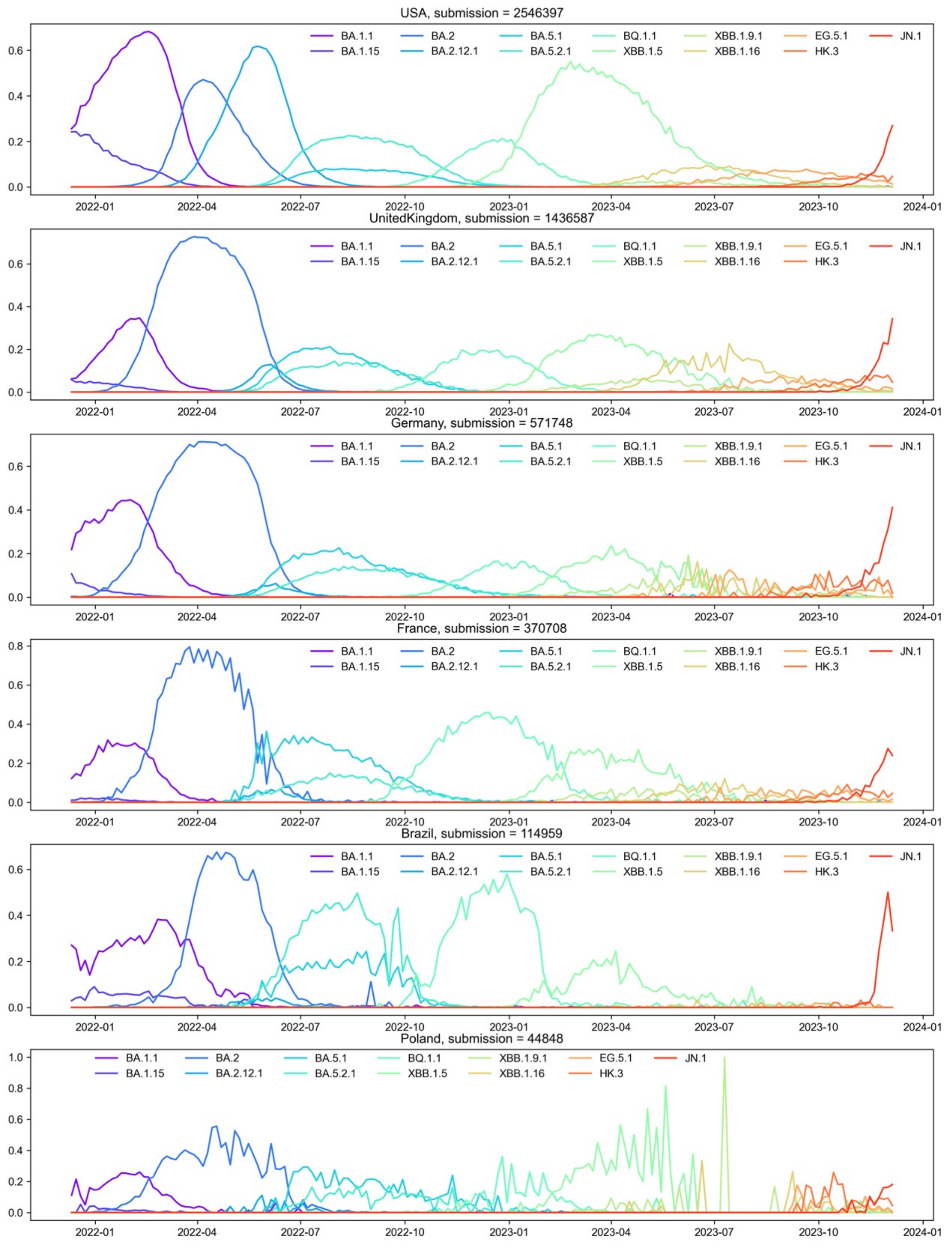

USA, submission = 2546397
BA.1.1
BA.1.15
BA.2
BA.2.12.1
BA.5.1
BA.5.2.1
BQ.1.1
XBB.1.5
XBB.1.9.1
XBB.1.16
EG.5.1
HK.3
JN.1
0.0
0.2
0.4
0.6
2022-01
2022-04
2022-07
2022-10
2023-01
2023-04
2023-07
2023-10
2024-01
UnitedKingdom, submission = 1436587
Germany, submission = 571748
France, submission = 370708
0.8
Brazil, submission = 114959
Poland, submission = 44848
1.0
