# Supplementary material for: Integrating genomic epidemiology and deep mutational scanning data for prevalence forecasting of SARS-CoV-2 Omicron lineages
Source: PLoS One. 2025 Nov 3;20(11):e0335520. doi: 10.1371/journal.pone.0335520 (PMC12582474; doi:10.1371/journal.pone.0335520)
Supplement: S19 Fig — (A) First-layer MLP weights across models. Heatmaps of the first-layer weight matrices (fc1; 100 × 200) for CoVPF and the three independently randomized-DMS controls (CMranDMS_ver1-ver3). (B) Pairwise Pearson correlation coefficients between the first-layer MLP weight matrices for CoVPF and CMranDMS_ver1-ver3. (PDF) [file pone.0335520.s019.pdf]

A

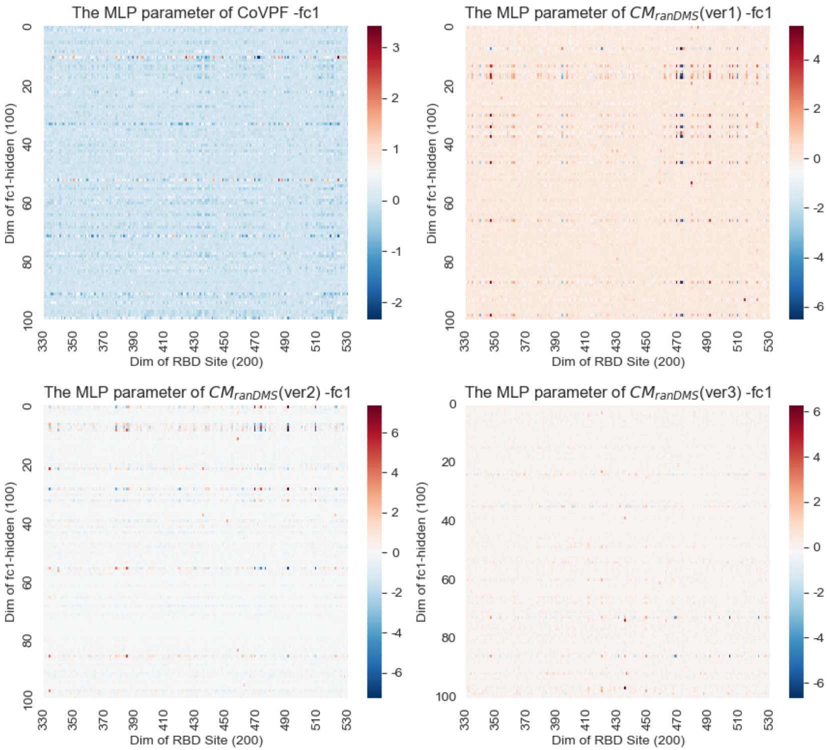

B

PCC	CoVPF	CMranDMS_ver1	CMranDMS_ver2	CMranDMS_ver3
CoVPF	\	\	\	\
CMranDMS_ver1	-0.00108	\	\	\
CMranDMS_ver2	-0.00469	-0.00749	\	\
CMranDMS_ver3	-0.00348	0.00588	-0.02931	\
