# Supplementary material for: Integrating genomic epidemiology and deep mutational scanning data for prevalence forecasting of SARS-CoV-2 Omicron lineages
Source: PLoS One. 2025 Nov 3;20(11):e0335520. doi: 10.1371/journal.pone.0335520 (PMC12582474; doi:10.1371/journal.pone.0335520)
Supplement: S12 Fig — (A) Fitness analysis of global Omicron lineages whose submissions > 10,000. The x axis represents the time of emergence, the y axis represents CoVPF fitness estimates, and the scatter size represents the scale of submission. Red scatters are non-recombinant lineages, blue scatters are recombinant lineages. (B) Distribution of the difference between regression values and fitness after regressing non-recombinant and recombinant lineages with submissions > 10,000 using linear regression. (C) Kernel density distributions of non-recombinant and recombinant lineages. Kolmogorov-Smirnov (K-S) test, p = 0.11, non-recombinant and recombinant lineages whose submissions > 1,0000 have the same distribution. Fitness analyses of lineages with > 5,000 submissions globally. (D) Relationship between lineages’ emergence time and fitness whose submissions > 5,000. € Distribution of the difference between regression values and fitness after regressing non-recombinant and recombinant lineages with submissions > 5,000 using linear regression. (F) Kernel density distributions of non-recombinant and recombinant lineages whose submissions > 5,000. Kolmogorov-Smirnov (K-S) test, p = 0.051, non-recombinant and recombinant lineages have the same distribution. (PDF) [file pone.0335520.s012.pdf]

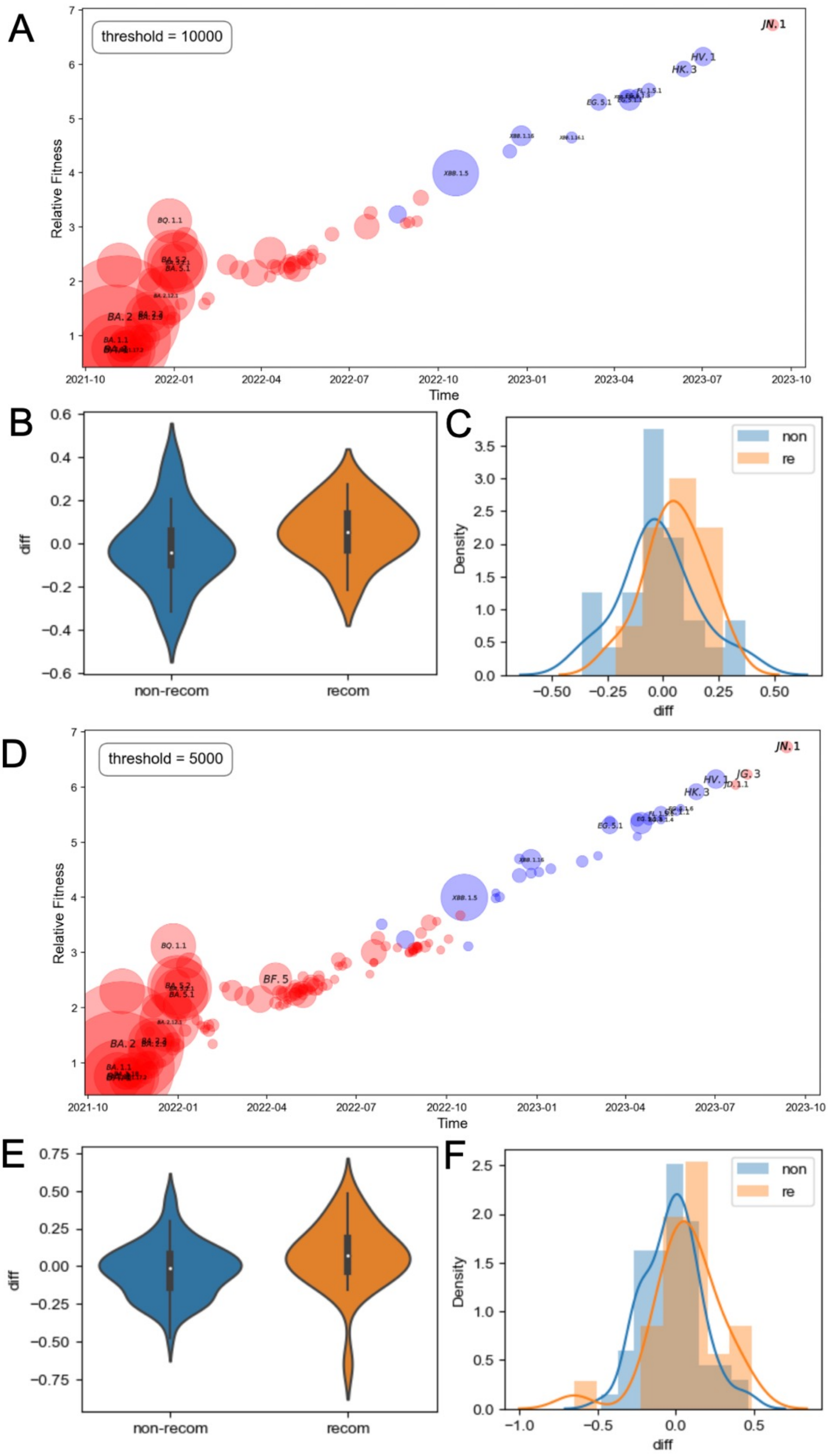

A
threshold = 10000
Relative Fitness
Time
2021-10
2022-01
2022-04
2022-07
2022-10
2023-01
2023-04
2023-07
2023-10
JN.1
HV.1
HK.3
EG.5.1
XBB.1.16
XBB.1.5
BQ.1.1
BA.5.2
BA.5.1
BA.2
BA.2.3
BA.1.1
B
diff
non-recom
recom
C
non
re
Density
diff
D
threshold = 5000
Relative Fitness
Time
JN.1
JG.3
HV.1
HK.3
EG.5.1
XBB.1.16
XBB.1.5
BQ.1.1
BF.5
BA.5.2
BA.5.1
BA.2
BA.2.3
BA.1.1
E
diff
non-recom
recom
F
non
re
Density
diff
